# Identification of Ischemic Stroke Patients Based on Plasma Concentrations of Extracellular Vesicles

**DOI:** 10.1007/s12975-025-01371-z

**Published:** 2025-08-15

**Authors:** Naomi C. Buntsma, Chi M. Hau, Mandy Los, Vivien M. Chen, Ton G. van Leeuwen, Yvo B. W. E. M. Roos, Rienk Nieuwland, Aleksandra Gasecka, Edwin van der Pol

**Affiliations:** 1 Laboratory of Experimental Clinical Chemistry and Amsterdam Vesicle Center, Laboratory Specialized Diagnostics & Research, Department of Laboratory Medicine, Amsterdam UMC, University of Amsterdam, Meibergdreef 9, Amsterdam, The Netherlands; 2 Department of Neurology, Amsterdam UMC, University of Amsterdam, Meibergdreef 9, Amsterdam, The Netherlands; 3 Biomedical Engineering & Physics, Amsterdam UMC, University of Amsterdam, Meibergdreef 9, Amsterdam, The Netherlands; 4 Amsterdam Neuroscience, Neurovascular Disorders, Amsterdam, The Netherlands; 5 Amsterdam Cardiovascular Sciences, Atherosclerosis and Ischemic Syndromes, Amsterdam, The Netherlands; 6 Amsterdam Cardiovascular Sciences, Microcirculation, Amsterdam, The Netherlands; 7 Cancer Center Amsterdam, Imaging and Biomarkers, Amsterdam, The Netherlands; 8 Chair and Department of Cardiology, Medical University of Warsaw, Warsaw, Poland; 9 Department of Cardiology, Onze Lieve Vrouwe Gasthuis, Amsterdam, The Netherlands; 10 Concord Clinical School, Faculty of Medicine and Health, University of Sydney, Sydney Australia Haematology, Concord Hospital and ANZAC Research Institute, Sydney Local Health District, Sydney, Australia

**Keywords:** Acute cerebrovascular accident, Biomarkers, Extracellular vesicles, Flow cytometry, Stroke

## Abstract

Patients presenting with stroke symptoms suffer from either ischemic stroke, hemorrhagic stroke, transient ischemic attacks (TIA), or “stroke mimics,” which include benign headaches, epilepsy, and vestibular disorders. As ischemic and hemorrhagic stroke patients require different medical treatments, early identification of the underlying cause of symptoms is essential for tailored and urgent medical intervention. This study investigates whether extracellular vesicles (EVs), present in peripheral blood of patients presenting with stroke symptoms, can be used to identify patients with ischemic stroke. Blood was collected from 155 patients presenting with stroke symptoms in the emergency room and analyzed for EVs by flow cytometry (ethics approval number NL72929.018.20). The primary endpoint was to compare platelet EV concentrations between patients with (*n* = 66) and without (*n* = 89) ischemic stroke. Concentrations of EVs from both activated platelets and leukocytes were lower in patients presenting with ischemic stroke compared to other patients (*p* = 0.038 and *p* = 0.015, respectively). No significant differences in other EV types were observed. In addition, ischemic stroke patients were older and had a higher diastolic blood pressure compared to patients with other diagnoses. In a multivariable analysis, leukocyte EVs and diastolic blood pressure were independent indicators of ischemic stroke. To conclude, this study demonstrates that the plasma concentration of leukocyte EVs can be useful to identify ischemic stroke patients in an emergency setting.

**Supplementary Information:**

The online version contains supplementary material available at 10.1007/s12975-025-01371-z.

## Introduction

Stroke is one of the leading causes of disability worldwide, with about 15 million people suffering from stroke annually [1]. There are two major subtypes of stroke: ischemic and hemorrhagic stroke. Ischemic stroke is caused by a cerebrovascular occlusion, whereas hemorrhagic stroke is caused by a ruptured cerebral blood vessel. Although the underlying causes differ, the symptoms of patients presenting with ischemic or hemorrhagic stroke are often indistinguishable. This similarity of symptoms complicates the tailored and urgent medical treatment.

At present, patients presenting with stroke symptoms are immediately transported to the nearest hospital for a computed tomography (CT) scan. In case of an ischemic stroke caused by a cerebrovascular occlusion, either tissue plasminogen activator (tPA) is administered or intra-arterial thrombectomy is performed, which is a method available only in specialized stroke centers [2–4]. In case of a cerebral hemorrhage, next to blood pressure control and regulation of blood coagulation, patients may qualify for surgical clipping or endovascular coiling if a cerebral aneurysm is identified as the cause of the hemorrhage. In addition to ischemic or hemorrhagic stroke, patients can present with stroke symptoms but in reality suffer from a transient ischemic attack (TIA) or “stroke mimics,” such as benign headaches, epilepsy, or vestibular disorders.

Since ischemic and hemorrhagic stroke as well as stroke mimics all require different treatment, the ability to diagnose the underlying cause of stroke-like symptoms at an early stage is of utmost importance. Currently, no clinically applicable diagnostic test is available for differential diagnosis in home or ambulance settings. Hence, there is a quest for biomarkers, present in liquid biopsies such as blood, that can be used to speed up the appropriate medical intervention in patients with stroke-like symptoms. Especially biomarkers that can discriminate ischemic stroke from all other diagnoses in home or ambulance settings are of interest, as ischemic stroke patients benefit most from urgent medical interventions.

EVs are membrane-delimited particles that are released by probably all cell types. Their biochemical composition, cellular origin, and function reflect real-time changes in an individual’s health status. Several studies measured the concentration of EVs in plasma of stroke patients. However, these concentrations were compared to those in plasma from healthy individuals [5–10], which does not address the clinical reality. Moreover, previously published data are irreproducible as data are in arbitrary units and detection limits unreported [11, 12]. Here, we addressed these questions by investigating whether *ischemic* stroke patients can be identified in a clinical setting by measuring (reproducible) concentrations of EVs in their blood plasma.

## Methods

### Patient Inclusion

This observational study is a preliminary analysis of the study Circulating Nanotraces to Identify the Cause of Stroke (CINTICS), which aims to identify nanotraces, including small RNAs and extracellular vesicles (EVs), that can identify stroke and discriminate between ischemic and hemorrhagic stroke. The study was approved by the Ethical Review Board of Amsterdam UMC, location AMC, in combination with a deferred consent process (approval number NL72929.018.20). Patients were included from October 2020 to November 2022.

Patients aged ≥ 18 years who presented at the emergency room (ER) of Amsterdam University Medical Center (Amsterdam UMC), location Academic Medical Center (AMC), with stroke-like symptoms underwent a cerebral computed tomography (CT) scan. The observations of the CT scan along with clinical symptoms were used to identify and classify the underlying cause of symptoms into ischemic strokes, hemorrhagic strokes, transient ischemic attacks (TIA), and stroke mimics. Ischemic stroke patients were classified into three subgroups: having a large vessel occlusion, a small occlusion, or no occlusion. Large vessel occlusions were identified as an occlusion in either the internal carotid arteries, the M1, or the proximal M2 segments of the middle cerebral arteries or vertebral arteries. In case the CT scan showed an occlusion in one of the smaller cerebral arteries, patients were classified as having ischemic stroke due to a smaller occlusion. Patients were diagnosed with ischemic stroke with no occlusion in case a CT scan did not show an occlusion, but either the CT perfusion scan or clinical symptoms as assessed by a neurologist strongly supported the diagnosis of ischemic stroke.

**Fig. 1:**
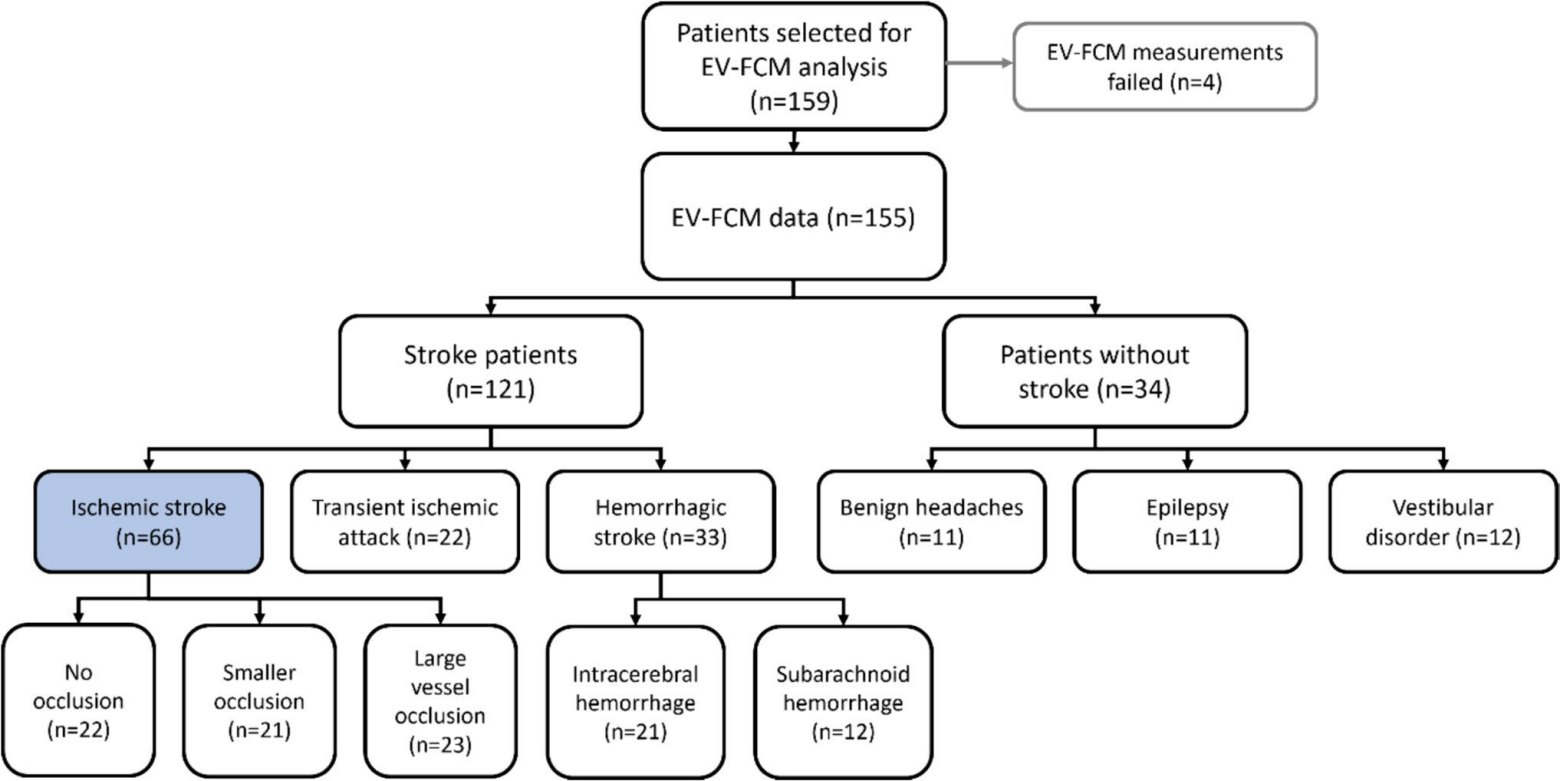
Flow chart of the study. The primary endpoint was to investigate differences in extracellular vesicle (EV) concentration between patients with ischemic stroke (blue) and all other patients. Secondary endpoints include differences in concentrations of other EV subtypes, as well as differences between other patients (sub)groups. *EV-FCM*, extracellular vesicle flow cytometry

Figure 1 shows the study flow chart. In this preliminary analysis, we had a capacity to analyze 150 to 160 samples. Based on power calculations, at least 64 ischemic stroke patients needed to be included. Stroke subtypes were balanced in number and matched for age and gender to the best of our ability, with the exception of subarachnoid hemorrhage, for which only 12 patients were present in the CINTICS database. Remaining patients were stroke mimics (*n* = 34) and were also balanced in number among three main subtypes and matched for age and gender to the best of our ability. Selection of patients was further based on the availability of at least four plasma aliquots in the biobank.

### Plasma Preparation

Blood was collected into tubes containing EDTA (4.0 mL K2EDTA, Becton Dickinson Vacutainer®, Franklin Lakes, NJ) as part of routine clinical care by antecubital venipuncture or from an intravenous drip. The tubes were transported from the ER to the laboratory within 2 h after collection. Plasma was prepared by double centrifugation as described earlier [13, 14]. The collected plasma was transferred to 1.5-mL Eppendorf tubes (Thermo Fisher Scientific, Waltham, MA), mixed by vortexing, and aliquots of 100 µL were stored at − 80 °C until analysis.

### Flow Cytometry Measurements

Concentrations of cell-type specific EVs were measured by flow cytometry (Northern Lights, Cytek Biosciences, Fremont, CA) after immunostaining. Concentrations of EVs from platelets (CD41^+^); activated platelets (P-selectin, CD62p^+^); erythrocytes (CD235a^+^); leukocytes (CD45^+^); monocytes and macrophages (CD14^+^); platelet endothelial cell adhesion molecule (PECAM-1, CD31^+^); epithelial cell adhesion molecule (EpCAM, CD326^+^); platelet cell death (using 4-[N-(S-glutathionylacetyl)amino]phenylarsonous acid, GSAO^+ ^[15, 16]) were determined.

To check for Fc-receptor binding, isotype controls were used. As a negative control for GSAO, 4-(N-[(S-glutathionyl)acetyl]amino)benzoic acid (GSCA) was used, because GSCA does not react with proteins but has the same biodistribution as GSAO [15]. Detailed information about the used antibodies and their concentrations can be found in the MIFlowCyt-EV, see Supplementary Material I.

Before labeling, samples were diluted in Dulbecco’s Phosphate Buffered Saline (DPBS) with a sample-specific dilution factor to ensure that the count rate during flow cytometry measurements was below 30,000 events/s, which prevents swarm detection on our instrument [17]. To label EVs, 2.5 µL of antibody was added to 20 µL of the diluted plasma and incubated for 2 h in the dark at room temperature. After incubation, 200 µL DPBS was added to decrease background fluorescence from unbound reagents.

Labelled samples were measured up to a volume of 150 µL or when a maximum of 10,000,000 counts was reached. On each measurement day, buffer-only controls, a positive control, and unstained controls were measured. The positive control was a stained pooled plasma sample. A volume of 30 µL was measured for the buffer-only and positive control. Unstained controls were measured at a higher dilution than the stained samples. Antibody dilutions were prepared weekly, and after each preparation, the buffer with reagents controls was performed.

Custom-built software (MATLAB R2020b, MathWorks, Natick, MA) was applied to automate data calibration and processing. Side scattering was related to the diameter of EVs in nm using Rosetta Calibration (v2.04, Exometry, The Netherlands), assuming that EVs have a core refractive index of 1.38, a shell refractive index of 1.48, and a shell thickness of 6 nm. Fluorescence signals were calibrated and expressed as units of molecules of equivalent soluble fluorochromes (MESF). EV concentrations represent the number of particles (i) exceeding the side scatter threshold, (ii) having a diameter between 100 and 1000 nm, and (iii) exceeding the fluorescent gate corresponding to the used labels, per mL of plasma. Data of EV subtypes resulting in < 100 counts on average was considered insignificant and therefore excluded from the analysis.

The experiments fulfill the criteria of the framework for standardized reporting of EV flow cytometry experiments [6]. MIFlowCyt-EV and datasheets are available via Figshare (see: 10.6084/m9.figshare.28688051).

### Statistical Analysis

As platelets are immediate response cells within the blood circulation and since platelets are associated with coagulation, inflammation, and occlusive thrombus formation, platelet-derived EVs may offer potential biomarkers since they can be measured in plasma. However, EVs originating from other cell types present within blood may also be discriminative and hence this study includes several EV types.

The primary endpoint was the difference in the plasma concentration of platelet EVs between patients with and without ischemic stroke. Ischemic stroke patients include those presenting with either large vessel occlusion (LVO) or ischemic stroke due to causes other than LVO (non-LVO).

Secondary endpoints include: (i) differences in plasma concentrations of other EV subtypes between patients with and without ischemic stroke and (ii) differences in plasma EV concentrations between patients with and without hemorrhagic stroke (intracerebral and subarachnoid hemorrhage), ischemic stroke due to a LVO, non-LVO ischemic stroke, transient ischemic attack (TIA), and stroke mimics.

As there is no data regarding the differences in EV concentrations in patients with and without different types of stroke, the power calculation for the primary endpoint was based on differences in EV concentrations in patients with ischemic stroke and healthy controls. Previously, we showed that patients with ischemic stroke have a twofold higher platelet EV concentration, compared to healthy volunteers [8]. The required sample size was calculated by a two-sided *t*-test at a significance level of 0.05 with the following assumptions: (i) mean nominal difference between the groups with and without ischemic stroke = 1.0, (ii) standard deviation (SD) ± 2.0, and (iii) nominal test power = 0.8. Based on this calculation, at least 64 patients with ischemic stroke should be included to observe a statistically significant difference in platelet EV concentrations in patients with or without ischemic stroke.

Distribution of data was checked using the Shapiro–Wilk test. Continuous variables were presented as mean with standard deviation or median with interquartile range, for normal and non-normal distributed data, accordingly. Comparison between continuous variables and EV concentrations were done using *t*-tests or Wilcoxon tests, according to distribution. Clinical data and categorical variables are presented as percentage of patients and were compared using *Χ*^2^ tests. In case EV concentrations were measured in duplicate, the average value was used for statistical analysis. The diagnostic value of EVs for ischemic stroke (primary endpoint) and the cut-off values were calculated using a receiver operating characteristic (ROC) curve. Logistic regression model incorporating EV concentrations and clinical characteristics which diagnosed ischemic stroke at *p* < 0.05 in the univariable analysis were included in a multivariable regression analysis. The results of both univariable and multivariable regression analyses are reported as odds ratio (OR) and 95% confidence interval (CI). A two-sided *p*-value < 0.05 was considered significant.

Statistical analyses were performed using SPSS Statistics version 27 (IBM, Chicago, IL, USA) and GraphPad Prism version 10.2.0 (GraphPad Software, San Diego, CA, USA).

## Results

Patient characteristics are shown in Table 1. Ischemic stroke patients (*n* = 66) were older (mean age 69.6 vs 64.7 years, *p* = 0.019) and had a higher diastolic blood pressure (DBP; mean 93 mmHg vs 87 mmHg, *p* = 0.019) than patients with other diagnoses (*n* = 93). There were no differences regarding other baseline characteristics, comorbidities, pharmacotherapy and laboratory data between patients with ischemic stroke, and other diagnoses.

**Table 1:** Comparison of baseline characteristics between patients who were diagnosed with ischemic stroke or other diagnoses. Unpaired *t*-tests or *U*-Mann–Whitney tests were used to compare normally and non-normally distributed data, respectively. Chi-square test was used to compare categorical variables. Ischemic stroke patients were older and had a higher diastolic blood pressure (DBP) than patients with other diagnoses. Since some patients were discharged or transferred to other hospitals before data collection was completed, there is some missing data in the dataset

	Total population (*n* = 155)	Ischemic stroke (*n* = 66)	Other diagnoses (*n* = 89)	*p*-value	Data complete
**Baseline characteristics**					
Age (years)	66.7 ± 13.1	69.6 ± 11.9	64.7 ± 13.6	**0.019**	100%
Gender (male)	75 (48.4%)	34 (51.5%)	41 (46.1%)	0.502	100%
BMI (kg/m^2^)	25.9 (23.2–29.5)	27.3 (22.1–33.0)	24.6 (23.5–29.2)	0.661	39%
SBP (mmHg)	157 ± 27	161 ± 23	157 ± 27	0.154	93%
DBP (mmHg)	89 ± 17	93 ± 18	87 ± 16	**0.019**	93%
**Comorbidities, *N* (%)**					
Prior stroke or TIA	40 (29.9%)	19 (30.2%)	21 (29.6%)	0.942	86%
Atrial fibrillation	22 (16.4%)	13 (20.6%)	9 (12.7%)	0.215	86%
Diabetes mellitus	30 (22.4%)	16 (25.4%)	14 (19.7%)	0.431	86%
Hypercholesterolemia	23 (17.2%)	10 (15.9%)	13 (18.3%)	0.709	86%
Hypertension	80 (59.7%)	40 (63.5%)	40 (56.3%)	0.399	86%
Coronary artery disease	18 (13.4%)	10 (15.9%)	8 (11.3%)	0.435	86%
Past or current smoking	54 (40.3%)	30 (47.6%)	24 (33.8%)	0.104	86%
Active cancer	15 (9.7%)	6 (11.3%)	9 (14.5%)	0.612	74%
**Pharmacotherapy, *N* (%)**					
Antiplatelet therapy	50 (32.3%)	25 (37.9%)	25 (28.1%)	0.197	98%
Vitamin K antagonist	7 (4.5%)	2 (3.0%)	5 (5.6%)	0.443	98%
DOAC	14 (9.0%)	7 (10.6%)	7 (7.9%)	0.556	98%
Antihypertensive drug	76 (50.0%)	29 (43.9%)	47 (54.7%)	0.191	98%
Statin	99 (66.0%)	37 (57.8%)	62 (72.1%)	0.068	97%
**Laboratory data**					
Platelets (*10^3^/µL)		249.0 (225.3–282.8)	255.0 (210.0–297.0)	0.723	97%
Leukocytes (*10^3^/µL)		8.0 (6.4–10.5)	8.3 (6.8–10.8)	0.438	97%
CRP (mg/L)		2.6 (0.8–7.6)	2.0 (0.8–5.6)	0.379	95%
Glucose (mg/dL)		7.1 (6.2–8.2)	7.1 (5.7–10.0)	0.978	95%
**Diagnosis, treatment, outcome *N* (%)**					
NIHSS (points)		5.0 (3.0–9.0)	0.0 (0.0–2.0)	< 0.001	43%
Occlusion on CT		43 (65.2%)	1 (1.1%)	< 0.001	100%
Reperfusion therapy	35 (22.6%)	33 (50.0%)	2 (2.3%)	< 0.001	100%
IVT	23 (65.7%)	21 (63.6%)	2 (100%)		
IAT	19 (54.3%)	19 (57.6%)	0 (0%)		
Mortality at 7 days	17 (11.1%)	4 (6.1%)	13 (14.9%)	0.083	99%

*BMI* body mass index, *CRP* C-reactive protein, *CT* computer tomography, *DBP* diastolic blood pressure, *DOAC* direct oral anticoagulant, *IAT* intra-arterial thrombectomy, *IVT* intravenous thrombolysis, *NIHSS* National Institutes of Health Stroke Scale, *TIA* transient ischemic attack, *SBP* systolic blood pressure

**Fig. 2:**
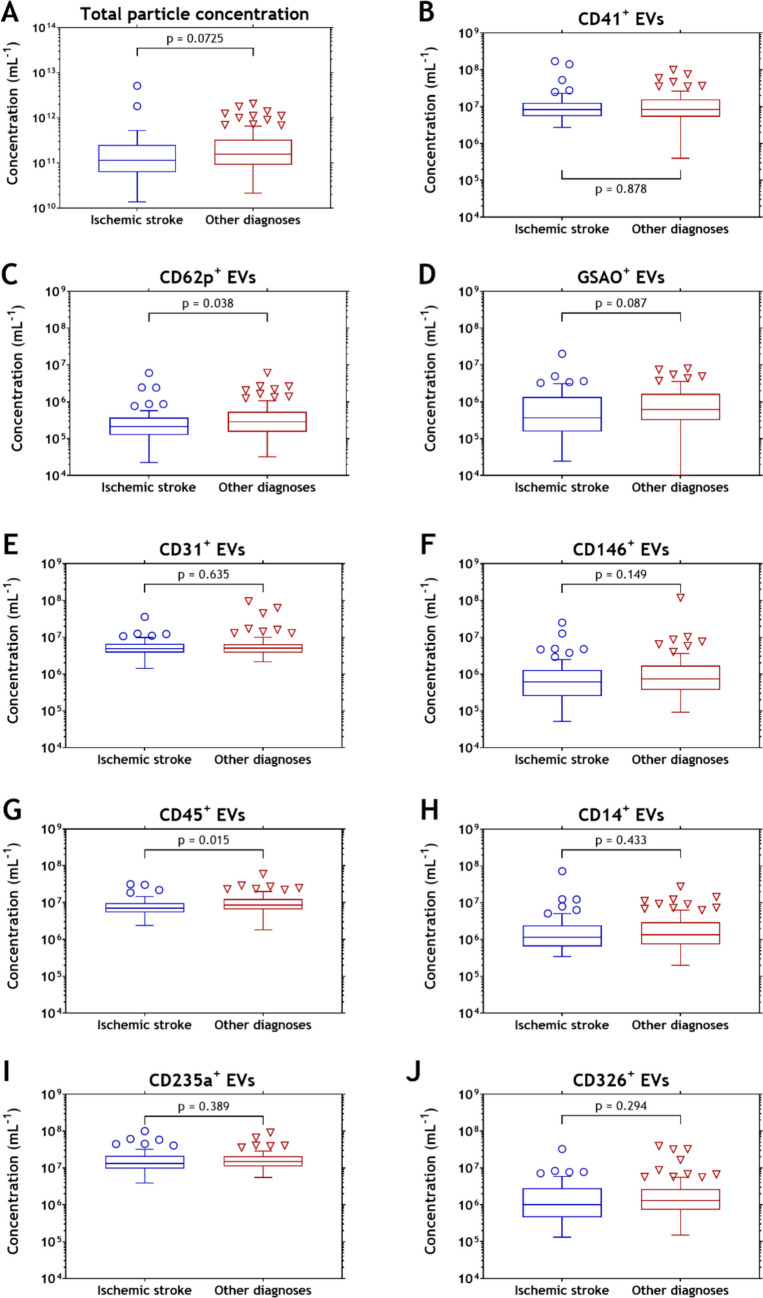
Plasma concentrations of extracellular vesicle (EV) subtypes in samples from patients with and without ischemic stroke. Panel A indicates the concentration of all particles measured during flow cytometry measurements. Panels B to H indicate concentrations of particles (i) with a diameter between 100 and 1000 nm and (ii) exceeding the fluorescent threshold corresponding to the used label (in molecules of equivalent soluble fluorophore or antibody binding capacity). Concentrations reflect the concentration of EVs derived from platelets (**B**, CD41^+^), activated platelets (**C**, CD62p^+^), death cells (**D**, GSAO^+^), platelet endothelial cell adhesion molecule (PECAM-1; **E**, CD31^+^), endothelial cells (**F**, CD146^+^), leukocytes (**G**, CD45^+^), monocytes and macrophages (**H**, CD14^+^), erythrocytes (**I**, CD235a^+^), and epithelial cell adhesion molecule (EpCAM; **J**, CD326^+^)

The measured concentrations of plasma EVs are summarized in Fig. 2. Figure 2C and G show that concentrations of EVs from activated platelets (CD62p^+^, *p* = 0.038) and leukocytes (CD45^+^, *p* = 0.015) are lower in plasma from ischemic stroke patients compared to patients without ischemic stroke. The concentrations of EVs from other (sub)types did not differ between patients with and without ischemic stroke (see Fig. 2).

**Fig. 3:**
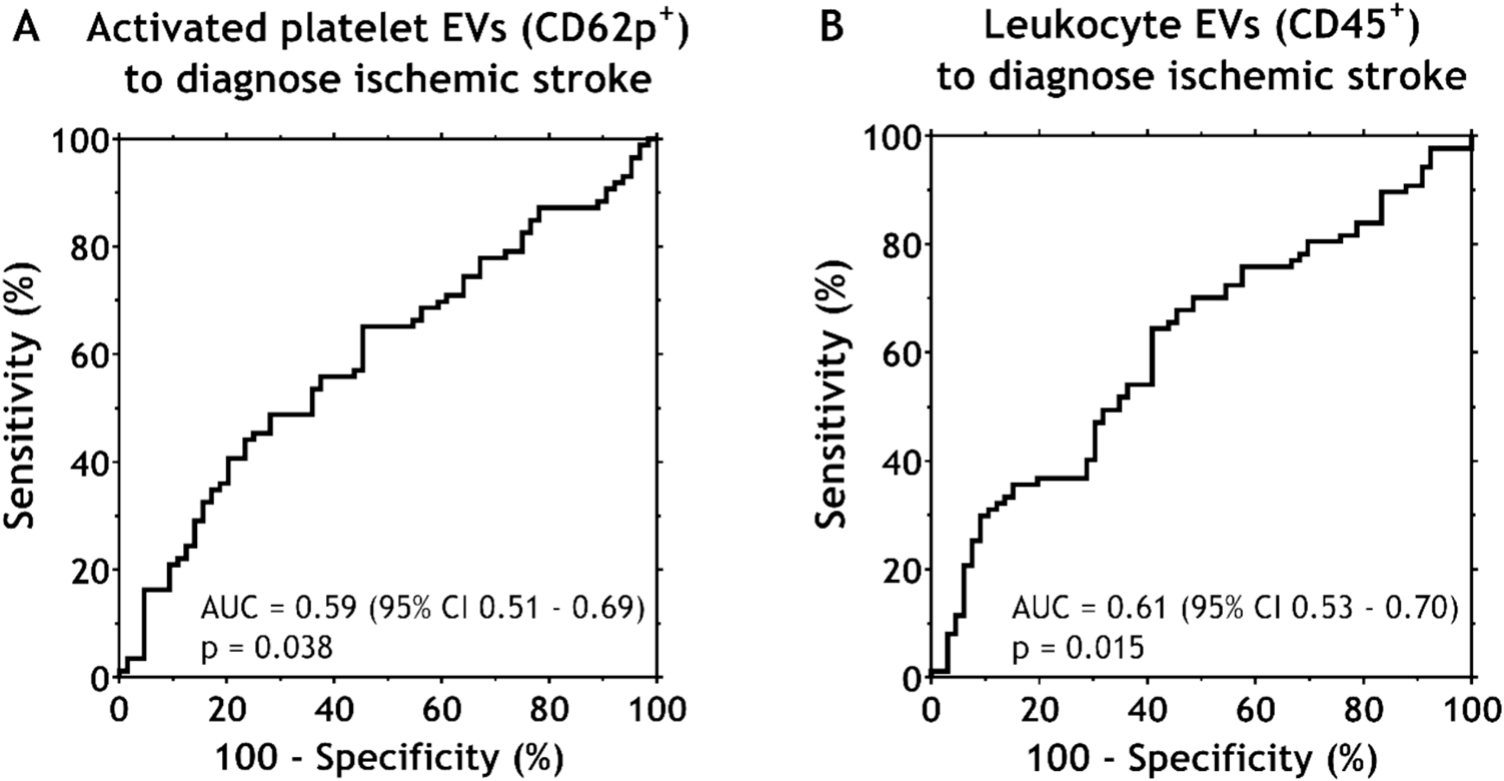
Receiver operating characteristic (ROC) curves to discriminate between patients diagnosed with ischemic stroke or an alternative diagnosis based on extracellular vesicles (EVs) derived from either activated platelets (CD62p^+^) or leukocytes (CD45^+^)

Figure 3 shows ROC curves to discriminate between patients diagnosed with ischemic stroke or other diagnoses based on the EV concentration from activated platelets (CD62p^+^, area under the curve (AUC) 0.60) and leukocytes (CD45^+^, AUC 0.61).

In univariable analysis (Table 2), ischemic stroke patients could be identified by age (53% sensitivity and 64% specificity); diastolic blood pressure (DBP, 71% sensitivity and 47% specificity); and the concentrations of EVs derived from activated platelets (CD62p^+^, 54% sensitivity and 66% specificity) and leukocytes (CD45^+^, 55% sensitivity and 67% specificity). To determine whether these factors are *independent* indicators of ischemic stroke, they were incorporated in multivariable analysis (Table 3). A leukocyte EV concentration below 7.71∙10^6^ mL^−1^ is associated with a threefold higher odds of ischemic stroke, independent of other variables (odds ratio (OR) 3.11, 95% confidence interval (CI) 1.35-7.16, *p* = 0.008). Moreover, patients with a DBP over 82 mmHg have fourfold higher odds of ischemic stroke, independent of other variables (OR 3.98, 95% CI 1.77-8.92, *p* = 0.001) The concentration of EVs derived from activated platelets and an age above 70 years are not independent indicators of ischemic stroke in multivariable analysis (*p* = 0.191 and *p* = 0.080, respectively).

**Table 2:** Statistical estimates to diagnose ischemic stroke based on age, diastolic blood pressure (*DBP*) and plasma concentrations at admission of extracellular vesicles (*EVs*) derived from activated platelets and leukocytes, based on receiver operating characteristic (*ROC*) analysis

	AUC (95% CI)	*p*-value	Cut-off	Sensitivity	Specificity	PPV	NPV
Age	0.61 (0.52–0.69)	0.026	> 70 years	53%	64%	52%	65%
DBP	0.61 (0.52–0.70)	0.022	> 82 mmHg	71%	47%	51%	68%
Activated platelet EVs	0.60 (0.51–0.69)	0.038	< 2.16 *10^5^ mL^−1^	54%	66%	55%	65%
Leukocyte EVs	0.61 (0.53–0.70)	0.015	< 7.71 *10^6^ mL^−1^	55%	67%	58%	64%

*AUC* area under the curve, *CI* confidence interval, *DBP* diastolic blood pressure, *EVs* extracellular vesicles, *PPV* positive predictive value, *NPV* negative predictive value

**Table 3:** Results of multivariable analysis to diagnose ischemic stroke using the concentrations of extracellular vesicles (*EVs*) derived from activated platelets and from leukocytes, along with clinical variables based on the cut-off values according to receiver operating characteristic (*ROC*) analysis. Both diastolic blood pressure (DBP) and the concentration of leukocyte EVs are independent indicators of ischemic stroke

	OR	95% CI		*p*-value
		Lower	Upper	
Age, > 70 years	1.93	0.93	4.01	0.080
DBP, > 82 mmHg	3.98	1.77	8.92	**0.001**
Activated platelet EVs, < 2.16 *10^5^ mL^−1^	1.69	0.77	3.68	0.191
Leukocyte EVs, < 7.71 *10^6^ mL^−1^	3.11	1.35	7.16	**0.008**

*DBP* diastolic blood pressure, *EVs* extracellular vesicles, *OR* odds ratio, *CI* confidence interval

**Fig. 4:**
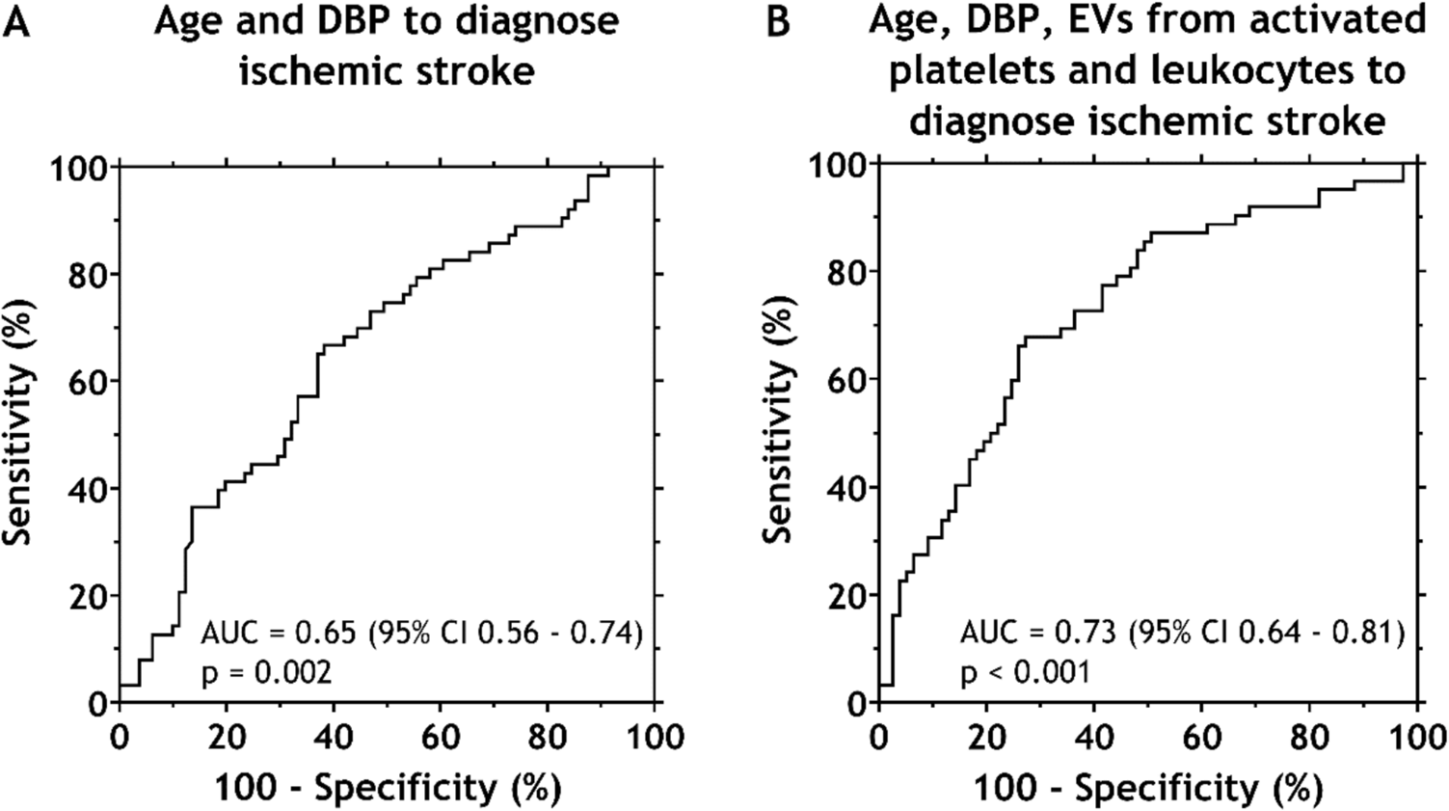
Receiver operating characteristic (ROC) curves to discriminate between patients diagnosed with ischemic stroke or an alternative diagnosis based on clinical variables only (**A**), or based on both clinical variables and the concentrations of extracellular vesicles (EVs) derived from activated platelets and leukocytes (**B**)

To assess the added diagnostic value of EV concentrations, we compared a multiple logistic regression model including only clinical variables (age and DBP) with a model that additionally incorporates EV concentrations from activated platelets and leukocytes. As shown in Fig. 4A, the model based on clinical parameters only yielded an AUC of 0.65. However, addition of EV concentrations increased the AUC to 0.73 (Fig. 4B), indicating improved diagnostic performance.

Plasma EV concentrations were also compared among other subgroups of patients. Patients with LVO had lower concentrations of leukocyte-derived EVs compared to all other patients (*p* = 0.021; see Supplementary Material II, Fig. 1). However, no difference was observed when comparing patients *without* LVO to all other patients (*p* = 0.397; see Supplementary Fig. 2). In contrast, the concentration of EVs from activated platelets (CD62p^+^) was lower in patients without LVO compared to all other patients (*p* = 0.045), but not in patients with LVO (*p* = 0.735). Hence, the LVO subgroup primarily accounts for the difference in leukocyte EV concentration between ischemic stroke patients and others, while the non-LVO subgroup contributes more significantly to the difference in the concentration of EVs from activated platelets.

Differences in plasma EV concentrations between patients with and without intracerebral hemorrhage, subarachnoid hemorrhage, TIA, and stroke mimics can be found in the Supplemental Materials (see Supplementary Material II, Figs. 3–6). Patients with a subarachnoid hemorrhage had higher concentrations of platelet EVs (CD41^+^) compared to all other patients (*p* = 0.017). There were no other differences between the groups.

## Discussion

In this study, we measured plasma concentrations of EVs in patients with stroke-like symptoms *before* the start of treatment. Our main findings are that (1) patients with ischemic stroke have lower plasma concentrations of EVs from activated platelets (CD62p^+^) and leukocytes (CD45^+^) compared to all other patients, (2) within the group of ischemic stroke patients, patients with an LVO have lower concentrations of leukocyte EVs (CD45^+^) than patients with non-LVO ischemic strokes, and (3) patients with a subarachnoid hemorrhage have higher concentrations of platelet EVs (CD41^+^) compared to all other patients.

Our conclusions are based on flow cytometry measurements of the concentrations of blood-derived plasma EVs, which are a balance between EV formation and clearance. Ischemic stroke results from thrombus formation in cerebral vessels, and involves activation of platelets, which results in adhesion, secretion, and aggregation. In addition, thrombus formation is characterized by leukocyte migration to the damaged vascular wall [18, 19]. These cellular processes may result in the release of EVs. It is possible that EVs from activated platelets and leukocytes accumulate at or near the damaged vascular wall, or in the thrombus itself, thereby resulting in a decrease in these EV concentrations in ischemic stroke patients. This possibility remains to be investigated, for example using a Chandler Loop system. Verification of this mechanism is essential, as our findings currently contrast with those of Vagida et al., who reported elevated concentrations of platelet-derived EVs in patients with acute coronary syndrome, which is also a thrombosis-related condition [20]. The reason for this discrepancy is unclear, but an incomplete removal of platelets and the use of an anticoagulant (citrate) that is unable to prevent the release of EVs from platelets after blood collection, are likely to explain the observed increase seen by Vagida et al. [21, 22]. Also, differences in handling of blood samples between patients and controls can explain differences in EV concentrations in previous studies [23].

In univariate analysis, both the concentrations of EVs from activated platelets and from leukocytes differed between ischemic stroke patients and other patients. Additionally, age and DBP were associated with ischemic stroke. Combining the concentrations of EVs from activated platelets and leukocytes with age and DBP improved the diagnostic accuracy in identifying ischemic stroke patients. In multivariable analysis, only leukocyte-derived EVs and DBP remained independent indicators of ischemic stroke. Aging increases the risk of developing cardiovascular events, including stroke. A relation between DBP and stroke incidence has been reported for both low and high DBP [24–26]. Low DBP could result in ischemic lesions since the mean arterial pressure drops beyond the range of autoregulation, thereby compromising cerebral blood flow [26, 27]. High DBP, on the other hand, may contribute to platelet activation and thrombi formation in atherosclerotic vessels due to turbulence [28, 29].

Within the group of ischemic stroke patients, the patients with LVO are unique because these patients can be treated in specialized hospitals where intra-arterial thrombectomy is performed. Thus, identification of LVO patients can be beneficial because these patients can then be transported immediately to the most suitable treatment facility. Dividing the ischemic stroke group into patients with and without LVO revealed that the concentration of leukocyte EVs was significantly lower in LVO patients compared to all other patients, while ischemic stroke patients without LVO had lower concentrations of EVs from activated platelets relative to all other patients. A study by Tarkanyi et al. [30] reported higher white blood cell (WBC) counts in LVO patients compared to patients without LVO. In our study, WBC counts did not differ (*p* = 0.634) but the concentration of leukocyte EVs (CD45^+^) was lower in LVO patients (*p* = 0.021) than in all other patients. However, since this was a subgroup analysis, these findings should be interpreted with caution.

As hemorrhagic stroke patients are typically treated by neurosurgeons, early identification of these patients would facilitate prompt involvement of the most appropriate specialists. When comparing the plasma EV concentrations of patients with intracerebral hemorrhages to all others, no differences were observed. Subarachnoid hemorrhages have disastrous downstream effects and require treatment by multidisciplinary teams [31]. Subarachnoid hemorrhage patients had higher concentrations of platelet EVs (CD41^+^) compared to other patients, which is in line with findings by Grossini et al., who compared EV concentrations in subarachnoid hemorrhage patients and healthy controls [32]. Again, considering the small sample size of the subgroup, this finding should be considered merely hypothesis-generating.

### Limitations

The limitations of this pilot study include (i) the relatively small sample size of certain subgroups, (ii) a low number of counts for some EV subtypes, and (iii) the gap between this pilot study and clinical implementation.

First, we selected samples from the CINTICS database. These samples were collected from patients *before* diagnosis, reflecting the diversity of people entering the ER with stroke-like symptoms—in fact, this is also a strength of our study. However, it also implies that patients were eventually diagnosed with either an ischemic stroke, hemorrhagic stroke, TIA, or a stroke mimic. To limit the variety in the group suffering a stroke mimic, we selected three common subdiagnoses: benign headaches, epilepsy, and vestibular disease. For adequate statistical analysis, especially in subgroup comparisons, the number of samples should be increased. Importantly, for our primary endpoint, i.e., identification of ischemic stroke patients, the number of samples was sufficient.

Second, some EV subtypes were measured but excluded because the measured number of positive EVs was low (on average, fewer than 100 measured particles between 100 and 1000 nm in the appropriate fluorescent range). Since the EV-containing plasma is diluted prior to measurement to reduce the risk of swarm detection [17], the measurement error increases because these low EV numbers are multiplied by the dilution factor, making the comparison of measured concentrations unreliable. In addition, state-of-the-art flow cytometers still lack the sensitivity to detect labeled EVs with a diameter below 100 nm. Although it is state-of-the-art to measure EVs down to 100 nm in a real clinical setting and in a reproducible manner, we still might detect only a few percent of all EVs [33, 34].

Third, although the current study demonstrates the potential of EV-based flow cytometry in plasma samples, both sample preparation and measurement remain time-consuming. As such, the method is not yet suitable for routine clinical application. However, efforts are taken to develop a point-of-care platform. The PHOREVER project (PHOtonic integrated OCT-enhanced flow cytometry for canceR and cardiovascular diagnostics enabled by Extracellular VEsicles discRimination) aims to design a multisensing platform with integrated pre-analytical steps [35]. This would represent a substantial step toward clinical implementation. Importantly, the EV biomarkers identified in the current pilot study could serve as a foundation for the development of a point-of-care test.

### Strengths

The present study also has multiple strengths. First, blood was collected in the ER, ensuring that our obtained results and data reflect real clinical conditions. Second, we compared ischemic stroke patients to all other patients presenting with stroke-like symptoms, rather than to healthy controls, which improves the clinical relevance of our findings. Third, our flow cytometry measurement data fulfil all current criteria and guidelines that support reproducibility and transparent reporting.

## Conclusions

This exploratory study assessed the diagnostic potential of plasma EVs to identify ischemic stroke patients. Concentrations of EVs from activated platelets and leukocytes are lower in plasma from ischemic stroke patients compared to all other patients who presented with similar symptoms. However, these concentrations alone are insufficient to reliably identify ischemic stroke patients. Including leukocyte EV concentration and diastolic blood pressure (DBP) in multivariable analysis may improve the diagnosis of ischemic stroke patients. Moreover, adding concentrations of EVs from activated platelets and leukocytes on top of age and DBP improves diagnostic accuracy, compared to a model based on only age and DBP. These findings suggest that blood-derived plasma EVs may be a potential biomarker that can be useful to identify ischemic stroke patients.

## Supplementary Information

Below is the link to the electronic supplementary material.

Supplementary file1 (PDF 377 KB)

Supplementary file2 (PDF 1.09 MB)

Supplementary file3 (PDF 688 KB)

## Data Availability

Data sheets can be found on Figshare, via https://doi.org/10.6084/m9.figshare.28688051.
