# Supplementary material for: Identification of Ischemic Stroke Patients Based on Plasma Concentrations of Extracellular Vesicles
Source: Transl Stroke Res. 2025 Aug 15;16(6):2082–92. doi: 10.1007/s12975-025-01371-z (PMC12596396; doi:10.1007/s12975-025-01371-z)
Supplement: Supplementary file 1 — Supplementary file1 (PDF 377 KB) [file 12975_2025_1371_MOESM1_ESM.pdf]

Supplementary Material I. MIFlowCyt-EV

'Identification of Ischemic Stroke Patients Based on Plasma Concentrations of Extracellular Vesicles'

1 Flow cytometry

1.1 Experimental design

The aim of flow cytometry (Northern Lights, Cytek Biosciences, Amsterdam) experiments was to measure the concentrations of extracellular vesicles (EVs) released from platelet/endothelial cells ($CD31^+$) , endothelial cells ($CD146^+$), leukocytes ($CD45^+$), erythrocytes ($CD235a^+$), macrophages ($CD14^+$), necrotic and late apoptotic cells ($GSAO^+$), platelets ($CD41^+$), activated platelets (P-selectin; $CD62p^+$), EPCAM ($CD326^+$) and lactadherin in platelet-depleted plasma (PDP) samples to compare EV concentrations in patients with stroke-like symptoms. We hypothesized that patients with an ischemic stroke could be differentiated from those without ischemic stroke. Patients without ischemic stroke were diagnosed with either (1) an ischemic stroke, (2) a transient ischemic attack (TIA), or (3) a stroke mimic, being epilepsy, benign headache or vestibular disease. We hypothesized that concentrations of EV types could differ according to diagnosis.

For this study, samples were measured on 24 different days between May 2023 and August 2023. All samples were measured using an autosampler, which facilitates subsequent measurements of samples in a 96-well plate.

To automatically apply calibrations, determine and apply gates, generate reports with scatter plots and generate data summaries, we used custom-build software (MATLAB R2020b, Mathworks, Natick, MA, USA).

1.2 Sample dilutions

To avoid swarm detection in typical PDP samples, we measured serial dilutions of five PDP samples with varying total particle concentrations to establish an approach on a different flow cytometer (Apogee A60-Micro, Apogee Flow Systems, UK). These experiments leaded to the conclusion that the combination of minimum dilution factor and a maximum count rate could prevent swarm detection in PDP samples measured with our settings at our flow cytometer[1].

By repeating this approach on the Northern Lights (Cytek Biosciences, Amsterdam) we found that swarm detection can be prevented in PDP samples when using a minimum dilution factor of about 100-fold and a maximum count rate of 30,000 events/s. To be on the safe side, we aimed for

---

[1] Buntsma et al. (2023) Preventing swarm detection in extracellular vesicle flow cytometry - a clinically applicable procedure. *Res Prac Thromb Heamost*.,

Corresponding author: Edwin van der Pol, e.vanderpol@amsterdamumc.nl

count rates ≤15,000 events/s for all measurements. Pre-staining, PDP samples were diluted 20- to 8,000-fold, followed by an additional 11.375-fold dilution post-staining, resulting in count rates between 1,501 and 61,299 events per second.

Measurements with count rates between 5,000 and 30,000 events/s were included in our study, while measurements with count rates below 5,000 or above 30,000 events/s were excluded.

1.3 EV staining

EVs were stained with antibodies. Prior to staining, antibodies were diluted in DPBS and centrifuged at 18,890 g for 5 min to remove aggregates. Supernatant, leaving at least 10 µL behind, was transferred to prepare antibody cocktails. Table S3 shows an overview of the used reagents and antibody concentrations during staining. Samples were labelled with combinations of either i) CD31-APC and CD146-PE, ii) CD45-APC, CD235a-PE and CD14-PacificBlue, iii) GSAO-AlexaFluor, CD62p-PE and CD41-PacificBlue or iv) CD326-APC and lactadherin-FITC. To stain, 20 µL of pre-diluted PDP was incubated with 7.5 µL of antibody or isotype cocktails. In case samples were stained with two antibodies, DPBS was added as a substitute for the third antibody. Samples were then kept in the dark for 2 h at room temperature. Post-staining, samples were diluted 11.375-fold in 200 µL of DPBS to decrease background fluorescence from unbound reagents.

1.4 Buffer-only control

Each measurement day at least 1 well with DPBS was measured with the same flow cytometer and acquisition settings as the samples. The mean count rate for buffer was 740.4 events per second, which is substantially lower than the count rates obtained for plasma samples (16,744.0 events per second).

1.5 Buffer with reagents control

Each 96-well plate contained a buffer with reagent control for each reagent (Table S3), which was measured with the same flow cytometer and acquisition settings as the samples. To investigate whether high background counts in buffer affected the reported results, we applied the same calibrations and gates to the reagents in buffer controls as to the stained samples.

Table S1 depicts the average number of positive counts per µL, obtained for buffer with reagent controls and for stained plasma samples. It was decided to include markers only when a) less than 0.5 events per µL were detected in buffer, and b) at least twice as many events were detected in samples compared to buffer. As can be noticed in Table S1, a high number of background events (high number of positive counts in buffer) was detected with lactadherin and hence these results were excluded from the manuscript.

Corresponding author: Edwin van der Pol, e.vanderpol@amsterdamumc.nl

Table S1. Average number of counts obtained per µL in buffer compared to stained samples.

	In buffer (evts/µL)	In samples (evts/µL)	Results ...
CD14-PacificBlue+	0.31	0.79	Included
CD31-APC+	0.05	3.67	Included
CD41-PacificBlue+	0.32	7.04	Included
CD45-APC+	0.13	5.13	Included
CD62p-PE+	0.02	0.18	Included
CD146-PE+	0.13	0.39	Included
CD235a-PE+	0.43	6.51	Included
CD326-APC+	0.22	0.62	Included
GSAO-AlexaFluor+	0.11	0.45	Included
Lactadherin-FITC+	29.20	51.18	Excluded

1.6 Isotype controls

Each day, isotype controls were added to one randomly selected plasma sample. The number of positive counts obtained with these controls, using the same settings and gates, is summarized in Table =S2. To ensure a fair comparison, the counts per µL in samples with isotypes controls were compared to those from the same plasma samples containing antibodies, as dilution factors in both are equal.

While a high number of positive counts was observed for some isotype controls – particularly for IgG-PE-, this was also accompanied by a similarly high count in buffer samples containing the isotype controls. This suggests a potential issue with the isotype control itself, which led to the decision not to exclude antibodies from further analysis.

Table S2. Number of counts obtained with isotype controls per µL in buffer compared to stained samples. Numbers indicate the median number.

Isotype control	With fluorescence thresholds of	Isotypes in plasma (evts/µL)	Antibodies in plasma (evts/µL)	Isotypes in buffer (evts/µL)
IgG1-APC	CD31-APC+	0.97	2.87	0.20
	CD45-APC+	0.90	7.67	0.20
	CD326-APC+	0.23	0.71	0.10
IgG1-PE	CD62p-PE+	9.07	0.08	4.52
	CD146-PE+	2.38	0.19	1.62
	CD235a-PE+	4.06	7.28	2.37
IgG1-PB	CD14-PacificBlue+	0.30	0.57	0.53
	CD41-PacificBlue+	0.30	4.30	0.50
GCSA	GSAO-AlexaFluor+	0.00	0.34	0.00

Corresponding author: Edwin van der Pol, e.vanderpol@amsterdamumc.nl

1.7 Trigger channel and threshold

Based on the buffer-only control, the acquisition software was set up to trigger at 1500 a.u. units SSC. The trigger was equivalent to an SSC cross section of 2 nm^2 and diameter of 99 nm (Rosetta Calibration, v2.04, Exometry, Amsterdam, The Netherlands).

1.8 Flow rate quantification

The Northern Lights is equipped with a flow rate sensor. Flow rate was set to be about 15 µL/min. Concentration measurements are based on the volume as determined by the flow rate sensor. In case the flow rate of a measurement deviated more than 2-fold from the anticipated 15 µL/min, the measurement was excluded from our analysis. Measurements were hence filtered on flow rates between 7.5 and 30.0 µL/min.

1.9 Fluorescence calibration

Calibration of the fluorescence detectors from arbitrary units (a.u.) to molecules of equivalent soluble fluorochrome (MESF) was accomplished using Quantitation APC beads (2364-19, BD), Quantitation FITC beads (2364-85, BD), Quantitation PE beads (2364-89, BD) and Quantum™ Alexa Fluor® 647 MESF beads (16510, Bangs Laboratories, Inc.). For PacificBlue we calibrated from arbitrary units (a.u.) to Antibody Binding Capacity (ABC) through the use of Quantum™ Simply Cellular® beads (16571, Bangs Laboratories, Inc.).

Calibrations were performed once per fluorescent detector. Rainbow beads (RCP-30-5A, Spherotech) were ran daily and cross-calibrated with the beads listed above to achieve daily calibration of fluorescent detectors. For each measurement, we added fluorescent intensities in MESF or ABC to the flow cytometry data files by custom-build software (MATLAB R2020b) using the following equation:

$$\mathrm{I(MESF)} = 10^{a \cdot \log_{10} \mathrm{I(a.u.)} + b} \qquad \text{Equation S1}$$

where I is the fluorescence intensity, and *a* and *b* are the slope and the intercept of the linear fits, respectively, see Table S4.

1.10 Light scatter calibration

We used Rosetta Calibration to relate scatter measured by SSC to the scattering cross section and diameter of EVs. We modelled EVs as core-shell particles with a core refractive index of 1.38, a shell refractive index of 1.48, and a shell thickness of 6 nm. For each measurement, we added the SSC scattering cross sections and EV diameter to the flow cytometry datafiles by custom-build software (MATLAB R2020a).

Corresponding author: Edwin van der Pol, e.vanderpol@amsterdamumc.nl

1.11 MIFlowCyt checklist

The MIFlowCyt checklist is added to Table S5.

1.12 EV number concentration

The concentrations reported in the manuscript describe the number of particles (1) that exceeded the SSC threshold of 1500 a.u., (2) that were measured with a flow rate between 7.5 and 30.0 µL/min, (3) with an average count rate between 5,000 and 30,000 events per second, (4) with a diameter <1,000 nm as measured by SSC after light scatter calibration and (5) positive for the fluorescence threshold (see Table S5, section 4.4.3), per mL of PDP.

1.13 Data sharing

Analyzed data sheets are available online (see https://doi.org/https://doi.org/10.6084/m9.figshare.28688051), data files can be requested.

Corresponding author: Edwin van der Pol, e.vanderpol@amsterdamumc.nl

Table S3: Overview of staining reagents. Characteristics being measured, analyte, analyte detector, reporter, isotype, clone, concentration, manufacturer, catalog number and lot number of used staining reagents.

Characteristic measured	Analyte	Analyte detector	Reporter	Isotype	Clone	Manufacturer	Catalog number	Lot number	Concentration used for staining (µg mL^{-1})
Myeloid differentiation	CD14	Anti-human CD14 antibody	PacificBlue	IgG1	63D3	Biolegend	367122	B354015	8.0
Platelet endothelial cell adhesion molecule	CD31	Anti-human CD31 antibody	APC	IgG1	WM59	Biolegend	303116	B302052	1.25
Integrin IIb, Megakaryocytic lineage	CD41	Anti-human CD41	PacificBlue	IgG1	HIP8	Biolegend	303714	B368404	3.2
Leukocyte common antigen	CD45	Anti-human CD45	APC	IgG1	HI30	Biolegend	304037	B272158	1.0
P-selectin, Platelet activation	CD62p	Anti-human CD62p antibody	PE	IgG1	CLBThromb/6	Beckman Coulter	IM1759U	200055	3.125
Endothelial cell lineage	CD146	Anti-human CD146 antibody	PE	IgG1	P1H12	Biolegend	361006	B337876	10.0
Glycophorin A	CD235a	Anti-human CD235a antibody	PE	IgG1	JC159	Dako	R7078	41443752	10.0
Epithelial cell adhesion molecule	CD326	Anti-human CD326 antibody	APC	IgG1	HEA-125	Miltenyi	130-113-260	5221000391	Stock was 64-fold diluted
Affinity for Fc receptor	Fc receptor	IgG1	APC	n.a.	MOPC-21	Beckman Dickinson	554681		8.0
Affinity for Fc receptor	Fc receptor	IgG1	PE	n.a.	X40	Beckman Dickinson	345816		10.0
Affinity for Fc receptor	Fc receptor	IgG1	PacificBlue	n.a.	MOPC-21	Biolegend	981812	13383084	3.2
Phosphatidyl serine	Lactadherin		FITC	n.a.	n.a.		BLAC-FITC	KK0804	Stock 83
	GSAO		AF647	n.a.	n.a.				0.00675 mM
	GCSA		AF647	n.a.	n.a.				0.00675 mM

AF647: Alexa Fluor 647; APC: allophycocyanin; CD: cluster of differentiation; FITC: fluorescein isothiocyanate;

GCSA: chemical substance 4-[N-[(S-glutathionyl) acetyl]amino)benzoic acid;

GSAO: chemical substance 4-[N-(S-glutathionylacetyl)amino]phenylarsonous acid; IgG: immunoglobulin G; PE: phycoerythrin.

Corresponding author: Edwin van der Pol, e.vanderpol@amsterdamumc.nl

Table S4: Overview of fluorescence calibrations

	APC (R1-A)		PE (B4-A)		FITC (B2-A)		AF647 (R2-A)		PacificBlue (V3-A)	
**Date (dd.mm.yy)**	Slope	Intercept	Slope	Intercept	Slope	Intercept	Slope	Intercept	Slope	Intercept
**Calibration date**	1.307	-3.032	1.271	-2.587	1.126	-1.262	0.832	-0.291	1.106	-1.396
**24.05.23**	1,312	-3,022	1,274	-2,655	1,111	-1,298	0,841	-0,335	1,103	-1,376
**25.05.23**	1,308	-2,989	1,277	-2,673	1,114	-1,323	0,837	-0,314	1,107	-1,398
**30.05.23**	1,302	-2,990	1,274	-2,664	1,114	-1,330	0,833	-0,311	1,109	-1,411
**31.05.23**	1,308	-3,009	1,275	-2,663	1,112	-1,318	0,836	-0,305	1,108	-1,401
**01.06.23**	1,311	-3,017	1,273	-2,653	1,109	-1,306	0,838	-0,321	1,104	-1,380
**05.06.23**	1,307	-3,002	1,274	-2,657	1,111	-1,322	0,834	-0,306	1,108	-1,405
**06.06.23**	1,312	-3,016	1,272	-2,642	1,106	-1,294	0,837	-0,316	1,099	-1,349
**08.06.23**	1,306	-3,006	1,274	-2,651	1,111	-1,320	0,834	-0,301	1,107	-1,399
**14.06.23**	1,301	-2,984	1,274	-2,644	1,109	-1,312	0,831	-0,287	1,108	-1,405
**15.06.23**	1,309	-3,012	1,274	-2,640	1,107	-1,304	0,836	-0,308	1,105	-1,386

Corresponding author: Edwin van der Pol, e.vanderpol@amsterdamumc.nl

**20.06.23**	1,304	-2,978	1,272	-2,619	1,104	-1,282	0,833	-0,289	1,099	-1,351
**21.06.23**	1,309	-3,003	1,274	-2,632	1,105	-1,290	0,836	-0,310	1,101	-1,363
**22.06.23**	1,306	-2,987	1,275	-2,633	1,105	-1,291	0,833	-0,287	1,101	-1,364
**27.06.23**	1,307	-3,010	1,277	-2,645	1,110	-1,317	0,834	-0,305	1,107	-1,401
**28.06.23**	1,306	-2,998	1,276	-2,635	1,108	-1,300	0,834	-0,296	1,107	-1,402
**29.06.23**	1,308	-3,002	1,276	-2,637	1,107	-1,299	0,833	-0,294	1,107	-1,400
**04.07.23**	1,306	-2,984	1,276	-2,633	1,108	-1,300	0,836	-0,309	1,106	-1,396
**05.07.23**	See 04.07.23									
**06.07.23**	1,308	-3,006	1,276	-2,634	1,106	-1,299	0,837	-0,312	1,107	-1,399
**11.07.23**	1,305	-2,998	1,278	-2,640	1,106	-1,291	0,837	-0,313	1,107	-1,401
**26.07.23**	1,303	-2,982	1,285	-2,680	1,107	-1,305	0,835	-0,307	1,106	-1,395
**27.07.23**	1,306	-2,986	1,277	-2,628	1,105	-1,293	0,836	-0,304	1,107	-1,398
**31.07.23**	1,304	-2,978	1,276	-2,614	1,106	-1,289	0,835	-0,294	1,106	-1,396
**09.08.23**	1,304	-2,999	1,276	-2,619	1,106	-1,297	0,833	-0,298	1,106	-1,396

APC: allophycocyanin; FITC: fluorescein isothiocyanate; PE: phycoerythrin; AF647: AlexaFluor647

Corresponding author: Edwin van der Pol, e.vanderpol@amsterdamumc.nl

Table S5. MIFlowCyt checklist.

**Requirement**	
1.1. Purpose	To compare the concentrations of EVs in plasma from patients with stroke-like symptoms, diagnosed with either an ischemic stroke, hemorrhagic stroke, transient ischemic attack or a stroke mimic.
1.2. Keywords	Extracellular vesicles, flow cytometry, stroke
1.3. Experiment variables	Plasma from patients with a suspicion of stroke, eventually diagnosed with either i) an ischemic stroke or ii) a different diagnosis, including hemorrhagic stroke, transient ischemic stroke and stroke mimics.
1.4. Organization name and address	Amsterdam University Medical Center<br>Location Academic Medical Centre<br>Meibergdreef 9<br>1105 AZ Amsterdam<br>The Netherlands
1.5. Primary contact name and email address	Edwin van der Pol, e.vanderpol@amsterdamumc.nl
1.6. Date or time period of experiment	May 2023 – August 2023
1.7 Other relevant experiment information	Samples were measured on 24 different days.
2.1. Biological sample source description	Blood samples were collected (4.0 mL K2EDTA, Vacutainer BD) either via antecubital venepuncture or through intravenous access. Platelet-depleted plasma was prepared by double centrifugation (Hettich Zentrifugen, Tuttlingen, Germany). The centrifugation parameters were: 2,500 g, 15 minutes, 20 ˚C, no brake. For freeze-storage, samples were transferred to 0.5 mL micro tubes (Sarstedt AG & Co., Germany), and stored in -80˚C. Before staining, samples were thawed for approximately 300 seconds at 37 °C.
2.2. Sample characteristics	Platelet-depleted plasma (PDP) is expected to contain EVs, lipoproteins and proteins. Residual platelets might be present.
2.3. Sample treatment description	Plasma samples were thawed in a warm water bath, diluted and measured to determine the optimal dilution factor for each sample separately. Samples were diluted according the determined optimal dilution factor and stained.

Corresponding author: Edwin van der Pol, e.vanderpol@amsterdamumc.nl

	Please see section S1.3 for staining procedure.
3.1. Instrument manufacturer	Cytek Biosciences, Amsterdam
3.2. Instrument model	Northern Lights
3.3. Instrument configuration and settings	Samples were analysed on a Northern Lights flow cytometer, at a flow rate of about 15 µL/min. Northern Lights flow cytometer is equipped with a 405 nm laser (area scaling factor 1.07), 488 nm laser (area scaling factor 1.24) and 640 nm laser (area scaling factor 1.07). The trigger threshold was set at SSC 1500 a.u., corresponding to an SSC cross section of 2 nm^2, (Rosetta Calibration).<br>For all detectors, the peak area was analysed. The gains for both FSC and SSC were 2000. APC signals were collected on channel R1 (bandpass filter 652-669 nm, gain 3000). PE signals were collected with the B4 channel detector (bandpass filter 571-590 nm, gain 1250). FITC signals were collected on channel B2 (bandpass filter 516-533 nm, gain 2500). AF647 signals were detected on channel R2 (bandpass filter 669-687 nm, gain 2500). PacificBlue signals were detected on channel V3 (bandpass filter 451-466 nm, gain 1250).
4.1. Data files	Data sheets are available via https://doi.org/https://doi.org/10.6084/m9.figshare.28688051, data files can be requested.
4.2. Compensation description	No compensation was required because no fluorophore combinations were used that have overlapping emission spectra.
4.3. Data transformation details	Fluorescence detectors were calibrated (section S1.09). SSC was calibrated with Rosetta Calibration (section S1.10). The concentrations reported in the manuscript describe the number of particles that fulfil the gating criteria per mL.
4.4.1. Gate description	To automatically apply gates, generate pdf reports with scatter plots, and summarize the data in a table, custom-build software (MATLAB R2020b) was used. Please find below a description of the gates. Second, events with a diameter <1,000 nm as measured by SSC after light scatter calibration (section S1.10) were included. Events positive for used fluorophores, being APC, PE, PacificBlue or AlexaFluor, were included.
4.4.2. Gate statistics	The number of positive events was corrected for flow rate, measurement time and dilutions performed during sample

Corresponding author: Edwin van der Pol, e.vanderpol@amsterdamumc.nl

	preparation.
4.4.3. Gate boundaries	Boundaries were 55 MESF for CD31-APC and CD45-APC, 80 MESF for CD326-APC, 90 MESF for CD62p-PE, 113 MESF for CD146-PE, 85 MESF for CD235a-PE and 500 MESF for Lactadherin-FITC. The boundary for GSAO-AlexaFluor was 1300 MESF.<br>Fluorescence for PacificBlue was calibrated using antibody capture (ABC) beads and was reflected in units of ABC. Used boundaries were 280 ABC for CD14-PacificBlue and 300 ABC for CD41-PacificBlue.

a.u.: arbitrary units; EVs: extracellular vesicles; FSC: forward scattering; MESF: Molecules of Equivalent Soluble Fluorophore; PDP: platelet depleted plasma; SSC: side scattering.

Corresponding author: Edwin van der Pol, e.vanderpol@amsterdamumc.nl
