# Supplementary material for: Identification of Ischemic Stroke Patients Based on Plasma Concentrations of Extracellular Vesicles
Source: Transl Stroke Res. 2025 Aug 15;16(6):2082–92. doi: 10.1007/s12975-025-01371-z (PMC12596396; doi:10.1007/s12975-025-01371-z)
Supplement: Supplementary file 2 — Supplementary file2 (PDF 1.09 MB) [file 12975_2025_1371_MOESM2_ESM.pdf]

Supplementary Material II. Additional Figures

'Identification of Ischemic Stroke Patients Based on Plasma Concentrations of Extracellular Vesicles'

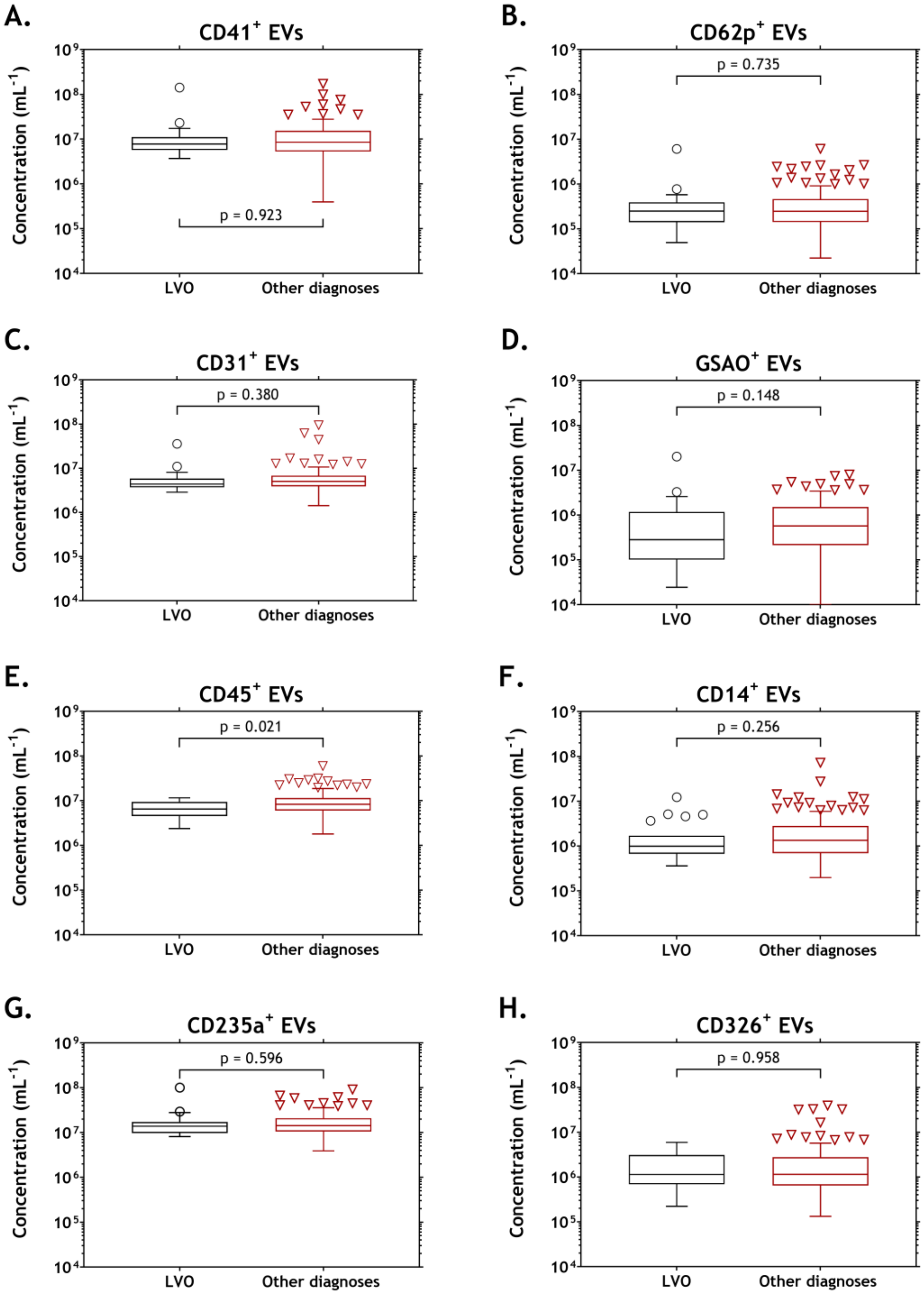

Supplementary Figure 1. Plasma concentrations of extracellular vesicle (EV) subtypes in samples from patients with and without large vessel occlusion (LVO) ischemic stroke. Panels indicate concentrations of particles i) with a diameter between 100 and 1,000 nm and ii) exceeding the fluorescent threshold corresponding to the used label (in molecules of equivalent soluble fluorophore or antibody binding capacity). Concentrations reflect the concentration of EVs derived from platelets (panel A, $CD41^+$), activated platelets (panel B, $CD62p^+$), platelet endothelial cell adhesion molecule (PECAM-1; panel C, $CD31^+$), death cells (panel D, $GSAO^+$), leukocytes (panel E, $CD45^+$), monocytes

and macrophages (panel F, CD14⁺), erythrocytes (panel G, CD235a⁺), and epithelial cell adhesion molecule (EpCAM; panel H, CD326⁺).

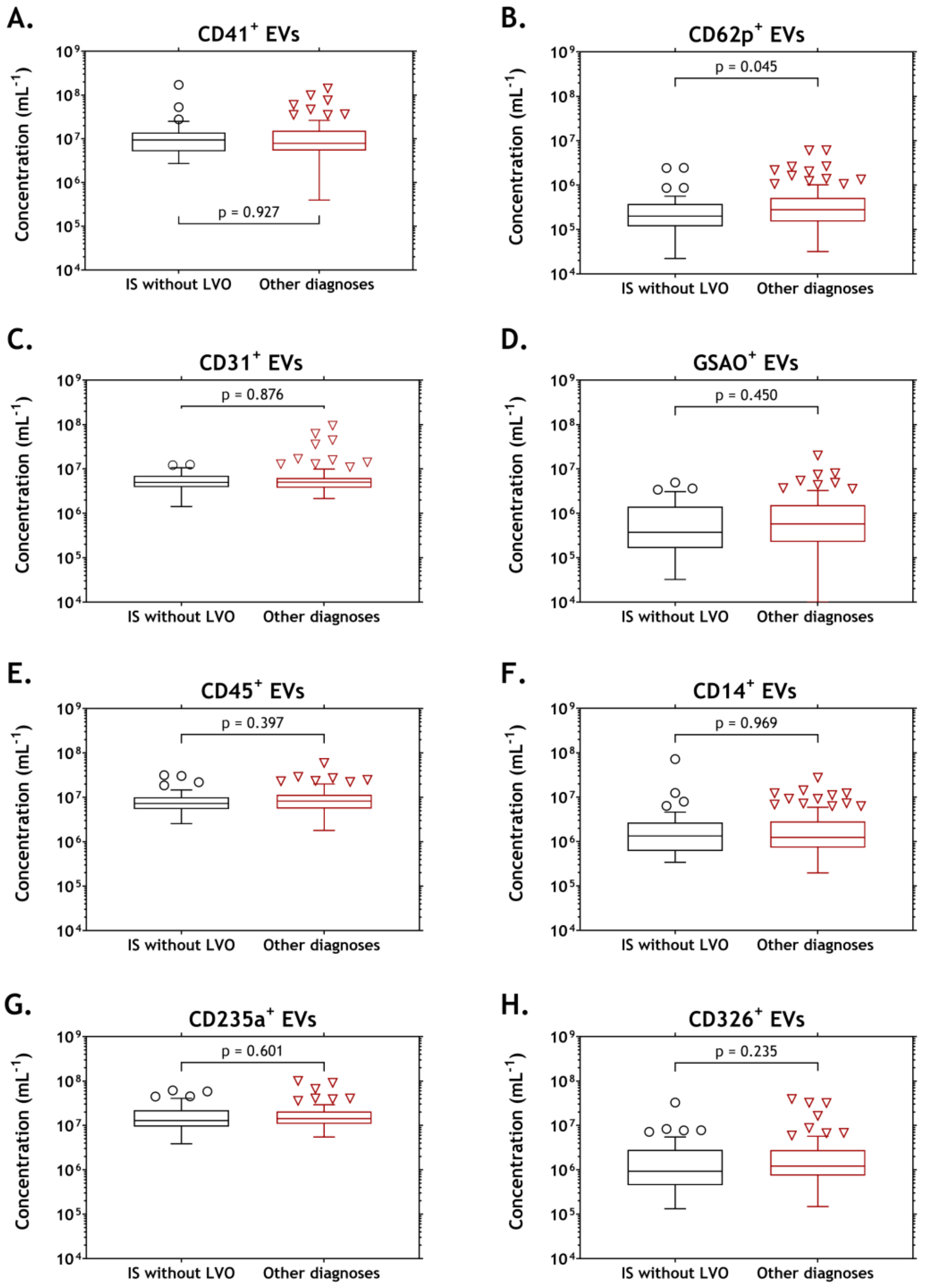

Supplementary Figure 2. Plasma concentrations of extracellular vesicle (EV) subtypes in samples from patients with an ischemic stroke (IS) nót due to a large vessel occlusion (LVO) compared to these measured in all other patients. Panels indicate concentrations of particles i) with a diameter between 100 and 1,000 nm and ii) exceeding the fluorescent threshold corresponding to the used label (in molecules of equivalent soluble fluorophore or antibody binding capacity). Concentrations reflect the concentration of EVs derived from platelets (panel A, CD41⁺), activated platelets (panel B, CD62p⁺), platelet endothelial cell adhesion molecule (PECAM-1; panel C, CD31⁺), death cells (panel D, GSAO⁺), leukocytes (panel E, CD45⁺), monocytes and macrophages (panel F, CD14⁺), erythrocytes (panel G, CD235a⁺), and epithelial cell adhesion molecule (EpCAM; panel H, CD326⁺).

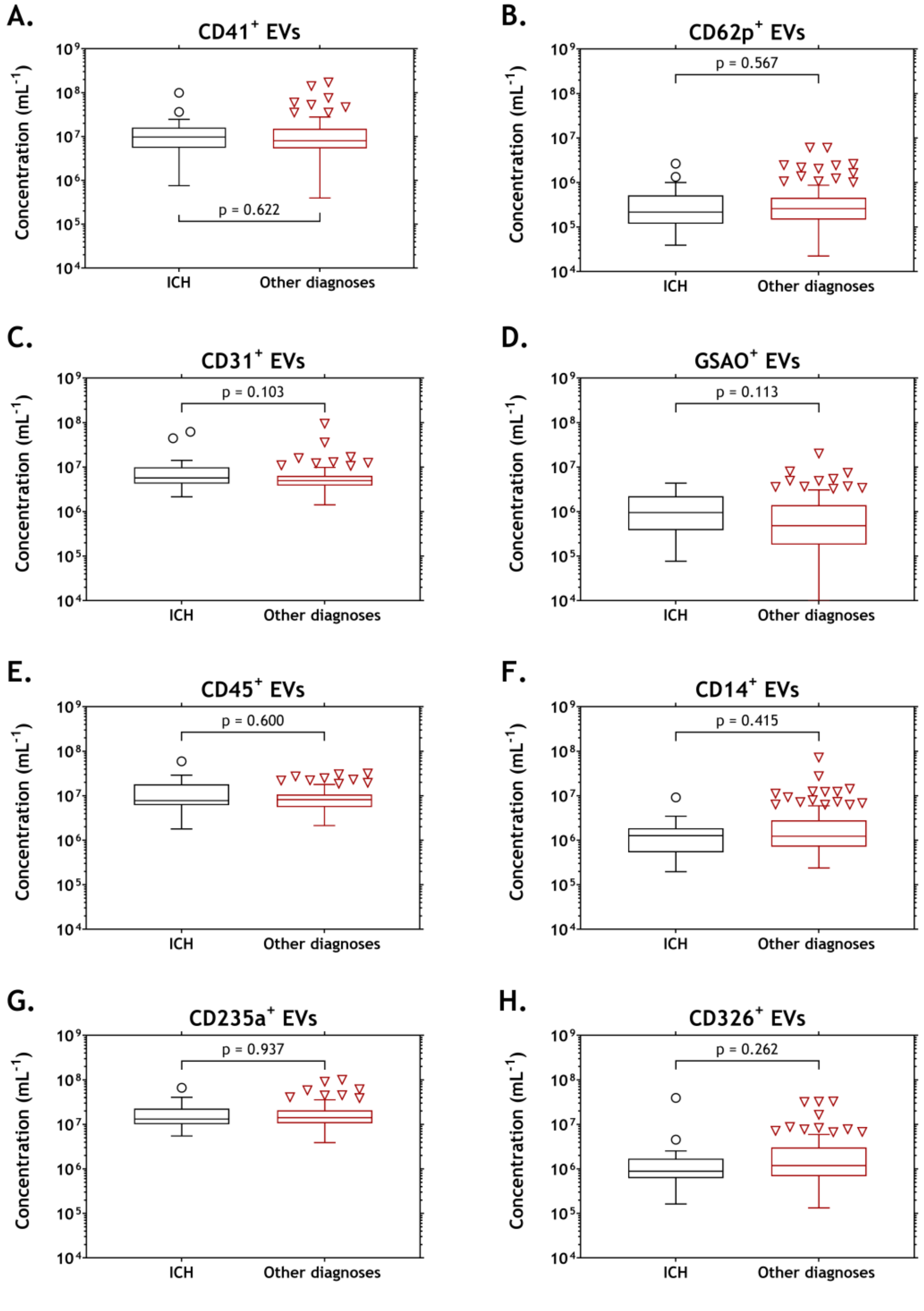

Supplementary Figure 3. Plasma concentrations of extracellular vesicle (EV) subtypes in samples from patients with and without intracerebral haemorrhage. Panels indicate concentrations of particles i) with a diameter between 100 and 1,000 nm and ii) exceeding the fluorescent threshold corresponding to the used label (in molecules of equivalent soluble fluorophore or antibody binding capacity). Concentrations reflect the concentration of EVs derived from platelets (panel A, CD41^{+}), activated platelets (panel B, CD62p^{+}), platelet endothelial cell adhesion molecule (PECAM-1; panel C, CD31^{+}), death cells (panel D, GSAO^{+}), leukocytes (panel E, CD45^{+}), monocytes and macrophages (panel F, CD14^{+}), erythrocytes (panel G, CD235a^{+}), and epithelial cell adhesion molecule (EpCAM; panel H, CD326^{+}).

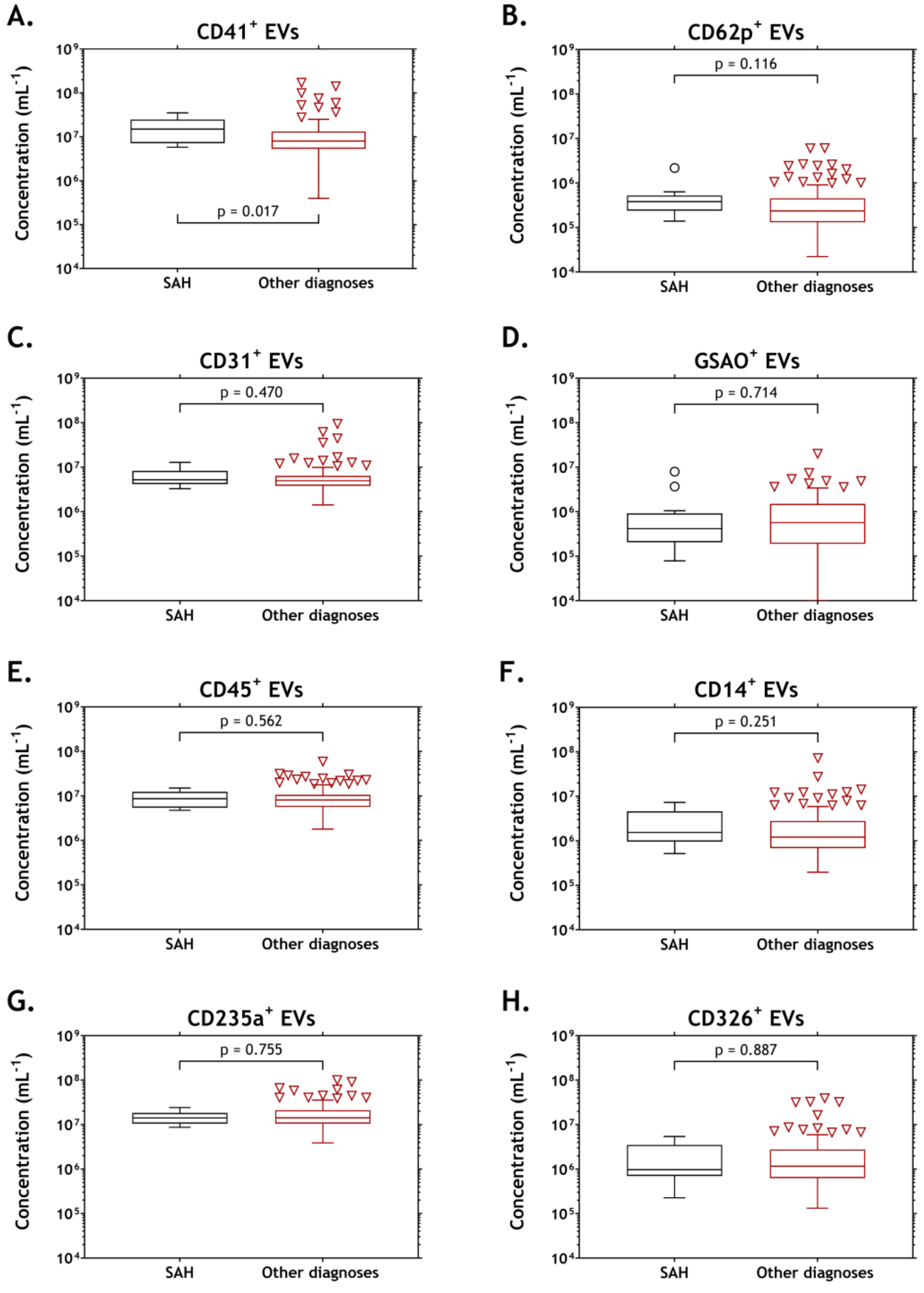

Supplementary Figure 4. Plasma concentrations of extracellular vesicle (EV) subtypes in samples from patients with and without subarachnoid haemorrhage (SAH). Panels indicate concentrations of particles i) with a diameter between 100 and 1,000 nm and ii) exceeding the fluorescent threshold corresponding to the used label (in molecules of equivalent soluble fluorophore or antibody binding capacity). Concentrations reflect the concentration of EVs derived from platelets (panel A, $CD41^+$), activated platelets (panel B, $CD62p^+$), platelet endothelial cell adhesion molecule (PECAM-1; panel C, $CD31^+$), death cells (panel D, $GSAO^+$), leukocytes (panel E, $CD45^+$), monocytes and macrophages (panel F, $CD14^+$), erythrocytes (panel G, $CD235a^+$), and epithelial cell adhesion molecule (EpCAM; panel H, $CD326^+$).

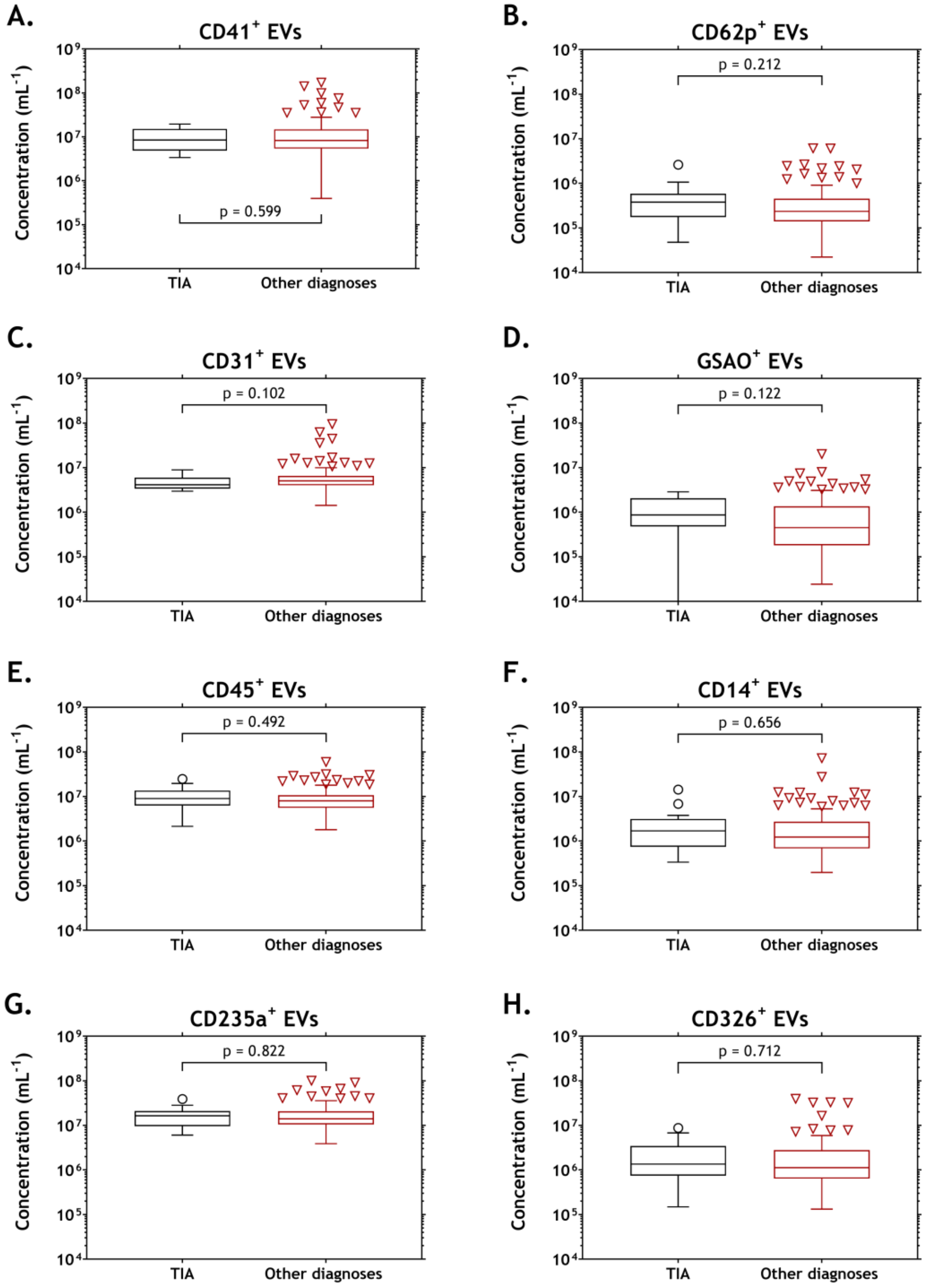

Supplementary Figure 5. Plasma concentrations of extracellular vesicle (EV) subtypes in samples from patients with and without transient ischemic attack (TIA). Panels indicate concentrations of particles i) with a diameter between 100 and 1,000 nm and ii) exceeding the fluorescent threshold corresponding to the used label (in molecules of equivalent soluble fluorophore or antibody binding capacity). Concentrations reflect the concentration of EVs derived from platelets (panel A, $CD41^+$), activated platelets (panel B, $CD62p^+$), platelet endothelial cell adhesion molecule (PECAM-1; panel C, $CD31^+$), death cells (panel D, $GSAO^+$), leukocytes (panel E, $CD45^+$), monocytes and macrophages (panel F, $CD14^+$), erythrocytes (panel G, $CD235a^+$), and epithelial cell adhesion molecule (EpCAM; panel H, $CD326^+$).

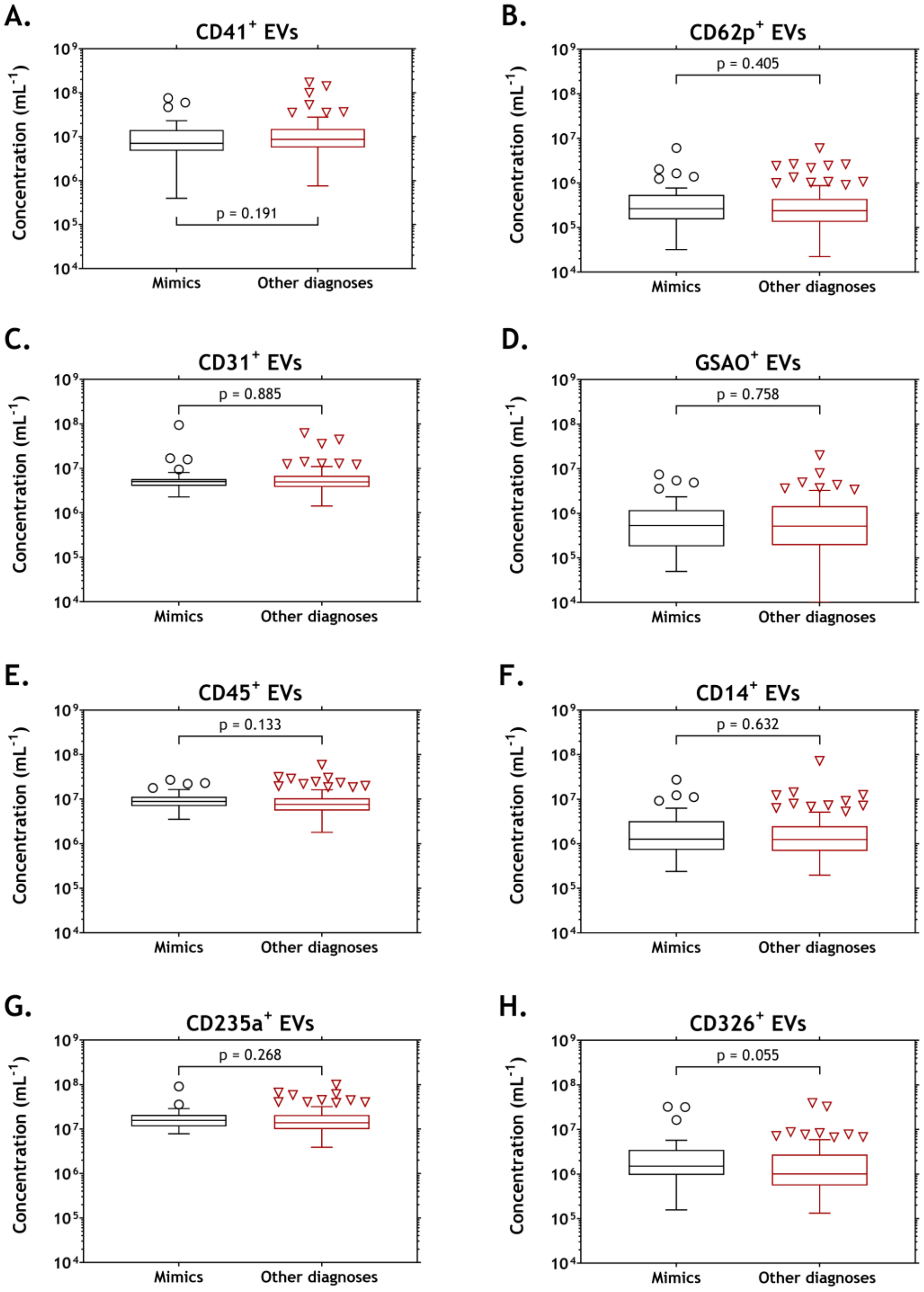

Supplementary Figure 6. Plasma concentrations of extracellular vesicle (EV) subtypes in samples from patients with and without stroke mimic. Panels indicate concentrations of particles i) with a diameter between 100 and 1,000 nm and ii) exceeding the fluorescent threshold corresponding to the used label (in molecules of equivalent soluble fluorophore or antibody binding capacity). Concentrations reflect the concentration of EVs derived from platelets (panel A, $CD41^+$), activated platelets (panel B, $CD62p^+$), platelet endothelial cell adhesion molecule (PECAM-1; panel C, $CD31^+$), death cells (panel D, $GSAO^+$), leukocytes (panel E, $CD45^+$), monocytes and macrophages (panel F, $CD14^+$), erythrocytes (panel G, $CD235a^+$), and epithelial cell adhesion molecule (EpCAM; panel H, $CD326^+$).
