# Supplementary material for: Identification of Ischemic Stroke Patients Based on Plasma Concentrations of Extracellular Vesicles
Source: Transl Stroke Res. 2025 Aug 15;16(6):2082–92. doi: 10.1007/s12975-025-01371-z (PMC12596396; doi:10.1007/s12975-025-01371-z)

Supplementary Material III. Gating Strategy

‘Identification of Ischemic Stroke Patients Based on Plasma Concentrations of Extracellular Vesicles’

Gating Strategy CD31-APC

Sample 4328

1. Removal of aggregates using a linear gate.

A linear line has been set to remove particles identified as aggregates by the reagents in buffer control.
The part indicated with an asterisk is included for further analysis.

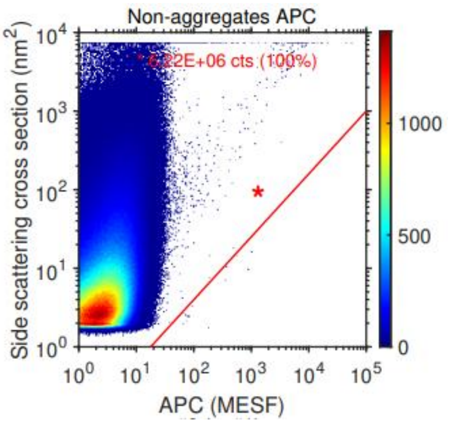

2. Removal of larger structures, including cells.

A gate has been set that excludes larger structures; with a large side scattering cross section and a high fluorescent signal.
The part indicated with an asterisk is included for further analysis.

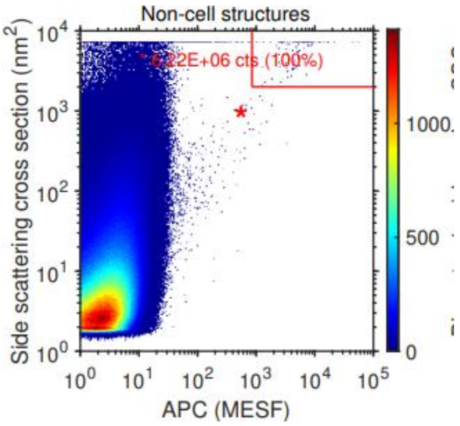

3. Selection of CD31-APC+ EVs.

Particles with a diameter between 100 and 1,000 nm, having a fluorescent signal that exceeds 55 MESF, were included as CD31-APC+ extracellular vesicles.

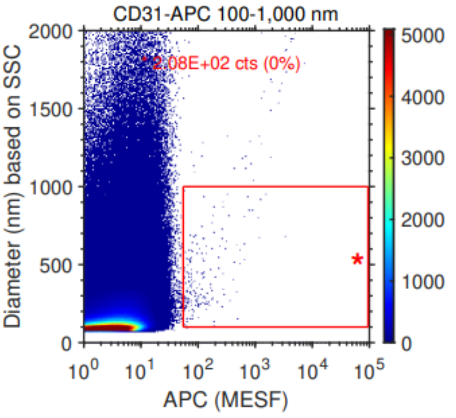

Gating Strategy CD146-PE

Sample 4328

1. Removal of aggregates using a linear gate.

A linear line has been set to remove particles identified as aggregates by the reagents in buffer control.
The part indicated with an asterisk is included for further analysis.

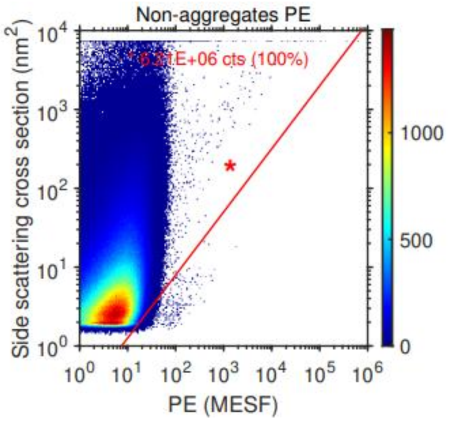

2. Selection of CD146-PE+ EVs.

Particles with a diameter between 100 and 1,000 nm, having a fluorescent signal that exceeds 113 MESF, were included as CD146-PE+ extracellular vesicles.

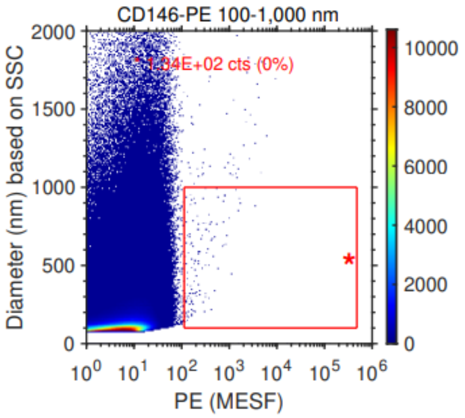

Gating Strategy CD45-APC

Sample 4328

1. Removal of aggregates using a linear gate.

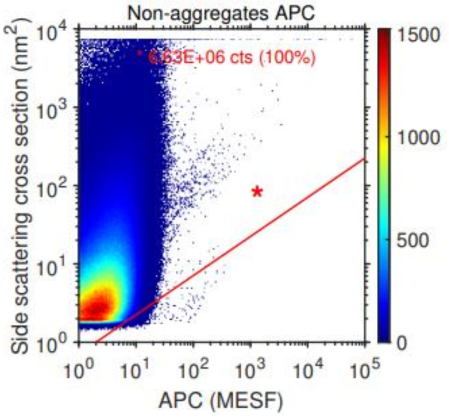

A linear line has been set to remove particles identified as aggregates by the reagents in buffer control.
The part indicated with an asterisk is included for further analysis.

2. Selection of CD45-APC+ EVs.

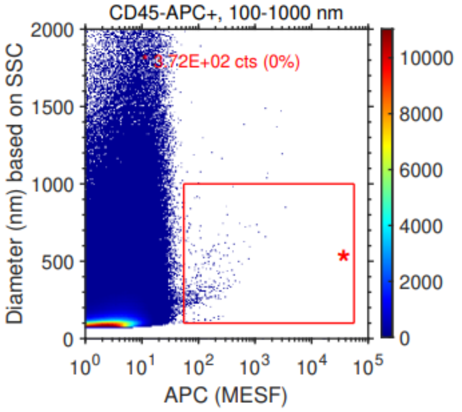

Particles with a diameter between 100 and 1,000 nm, having a fluorescent signal that exceeds 55 MESF, were included as CD45-APC+ extracellular vesicles.

Gating Strategy CD235a-PE

Sample 4328

1. Removal of aggregates using a linear gate.

A linear line has been set to remove particles identified as aggregates by the reagents in buffer control.
The part indicated with an asterisk is included for further analysis.

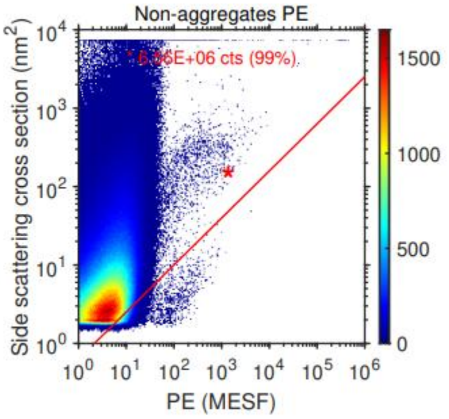

2. Selection of CD235a-PE+ EVs.

Particles with a diameter between 100 and 1,000 nm, having a fluorescent signal that exceeds 85 MESF, were included as CD235a-PE+ extracellular vesicles.

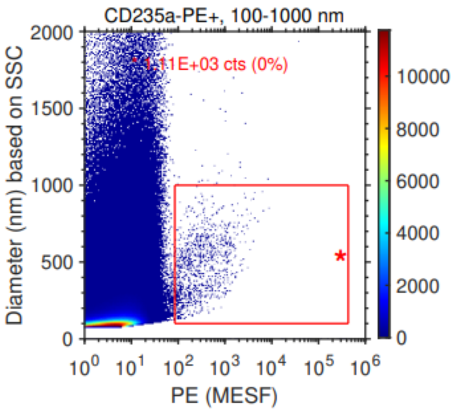

Gating Strategy CD14-PB

Sample 4328

1. Selection of CD14-PB+ EVs.

No subgates were applied.

Particles with a diameter between 100 and 1,000 nm, having a fluorescent signal that exceeds 280 ABC, were included as CD14-PB+ extracellular vesicles.

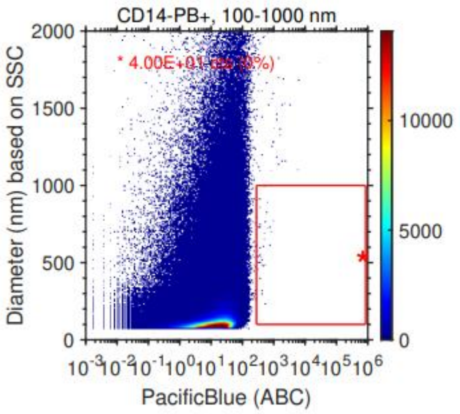

Gating Strategy CD326-APC

Sample 4328

1. Removal of aggregates using a linear gate.

A linear line has been set to remove particles identified as aggregates by the reagents in buffer control.
The part indicated with an asterisk is included for further analysis.

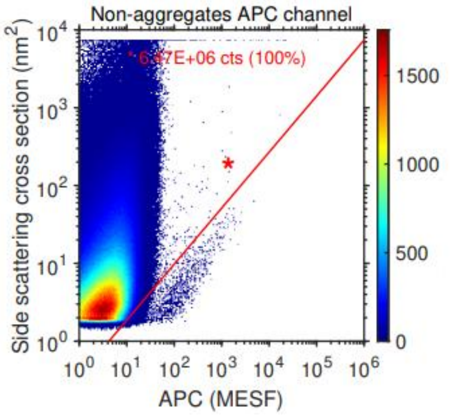

2. Selection of CD326-APC+ EVs.

Particles with a diameter between 100 and 1,000 nm, having a fluorescent signal that exceeds 80 MESF, were included as CD326-APC+ extracellular vesicles.

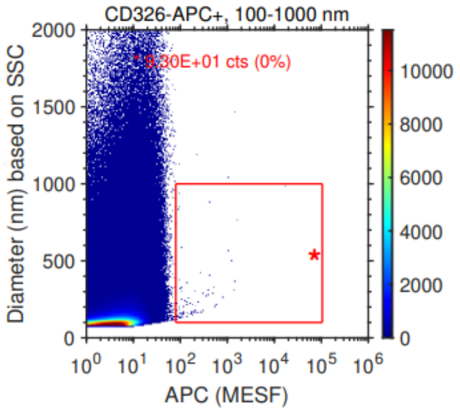

Gating Strategy GSAO-AF

Sample 4328

1. Removal of aggregates using a linear gate.

A linear line has been set to remove particles identified as aggregates by the reagents in buffer control.
The part indicated with an asterisk is included for further analysis.

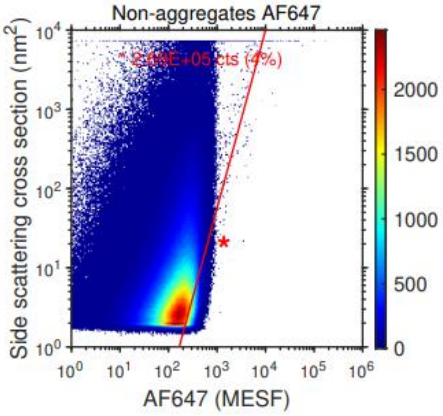

2. Selection of GSAO-AF+ EVs.

Particles with a diameter between 100 and 1,000 nm, having a fluorescent signal that exceeds 1300 MESF, were included as GSAO+ extracellular vesicles.

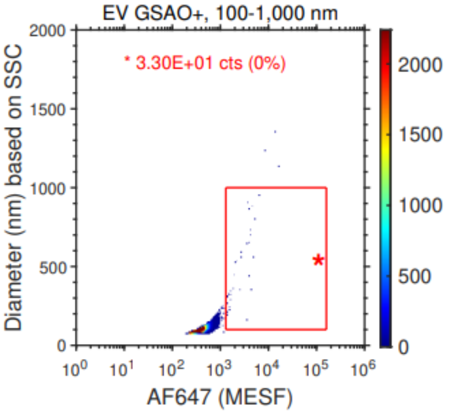

Additional example, sample 9055

1. Removal of aggregates using a linear gate.

A linear line has been set to remove particles identified as aggregates by the reagents in buffer control.
The part indicated with an asterisk is included for further analysis.

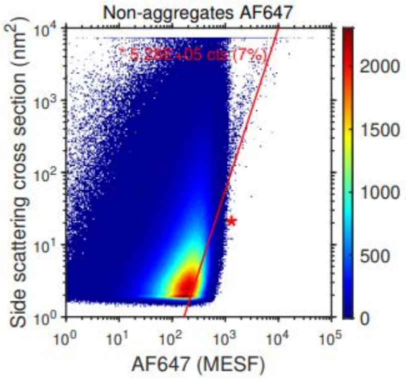

2. Selection of GSAO-AF+ EVs.

Particles with a diameter between 100 and 1,000 nm, having a fluorescent signal that exceeds 1300 MESF, were included as GSAO+ extracellular vesicles.

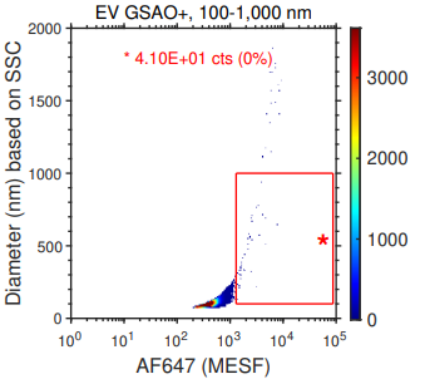

Gating Strategy CD62p-PE

Sample 4328

1. Selection of CD62p-PE+ EVs.

No subgates were applied.

Particles with a diameter between 100 and 1,000 nm, having a fluorescent signal that exceeds 90 MESF, were included as CD62p-PE+ extracellular vesicles.

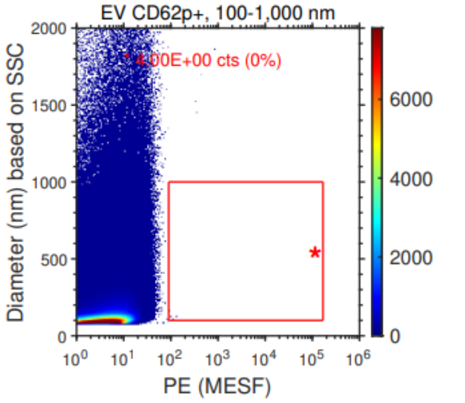

Gating Strategy CD41-PB

Sample 4328

1. Selection of CD41-PB+ EVs.

No subgates were applied.

Particles with a diameter between 100 and 1,000 nm, having a fluorescent signal that exceeds 300 ABC, were included as CD41-PB+ extracellular vesicles.

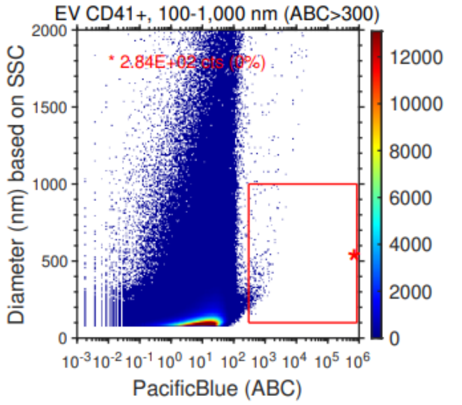

Supplement: Supplementary file 3 — Supplementary file3 (PDF 688 KB) [file 12975_2025_1371_MOESM3_ESM.pdf]
